# GPR84 signaling promotes intestinal mucosal inflammation via enhancing NLRP3 inflammasome activation in macrophages

**DOI:** 10.1038/s41401-021-00825-y

**Published:** 2021-12-15

**Authors:** Qing Zhang, Lin-hai Chen, Hui Yang, You-chen Fang, Si-wei Wang, Min Wang, Qian-ting Yuan, Wei Wu, Yang-ming Zhang, Zhan-ju Liu, Fa-jun Nan, Xin Xie

**Affiliations:** 1 State Key Laboratory of Drug Research, The National Center for Drug Screening, Shanghai Institute of Materia Medica, Chinese Academy of Sciences, Shanghai, 201203 China; 2 School of Pharmaceutical Science and Technology, Hangzhou Institute for Advanced Study, University of Chinese Academy of Sciences, Hangzhou, 310024 China; 3 University of Chinese Academy of Sciences, Beijing, 100049 China; 4 Department of Gastroenterology, The Shanghai Tenth People’s Hospital, Tongji University, Shanghai, 200072 China; 5 Burgeon Therapeutics Co., Ltd, Shanghai, 201203 China; 6 Yantai Key Laboratory of Nanomedicine and Advanced Preparations, Yantai Institute of Materia Medica, Yantai, 264000 China

**Keywords:** GPR84, medium chain fatty acid receptor, GPCR, inflammatory bowel diseases, ulcerative colitis, NLRP3 inflammasome, macrophages

## Abstract

The putative medium-chain free fatty acid receptor GPR84 is a G protein-coupled receptor primarily expressed in myeloid cells that constitute the innate immune system, including neutrophils, monocytes, and macrophages in the periphery and microglia in the brain. The fact that GPR84 expression in leukocytes is remarkably increased under acute inflammatory stimuli such as lipopolysaccharide (LPS) and TNFα suggests that it may play a role in the development of inflammatory and fibrotic diseases. Here we demonstrate that GPR84 is highly upregulated in inflamed colon tissues of active ulcerative colitis (UC) patients and dextran sulfate sodium (DSS)-induced colitis mice. Infiltrating GPR84^+^ macrophages are significantly increased in the colonic mucosa of both the UC patients and the mice with colitis. Consistently, GPR84^−/−^ mice are resistant to the development of colitis induced by DSS. GPR84 activation imposes pro-inflammatory properties in colonic macrophages through enhancing NLRP3 inflammasome activation, while the loss of GPR84 prevents the M1 polarization and properties of proinflammatory macrophages. CLH536, a novel GPR84 antagonist discovered by us, suppresses colitis by reducing the polarization and function of pro-inflammatory macrophages. These results define a unique role of GPR84 in innate immune cells and intestinal inflammation, and suggest that GPR84 may serve as a potential drug target for the treatment of UC.

## Abstract (web summary)

GPR84 is positively correlated with ulcerative colitis in patients. Deletion or blockade of GPR84 alleviates colitis in mice via reducing NLRP3 activation and M1 polarization

## Introduction

Inflammatory bowel diseases (IBDs), principally comprising Crohn’s disease (CD) and ulcerative colitis (UC), are chronic, relapsing, and remitting disorders of the gastrointestinal tract that cause progressive, sometimes irreversible, structural and functional damage of the bowel [1, 2]. The precise etiology of IBD remains obscure, but several factors including environment, genetic make-up, gut microbiota and immune response are believed to make a major contribution to the diseases. Dysregulation of immune responses and multiply of proinflammatory mediators are the two hallmarks of IBD that make it recurrent and nearly incurable [1, 3, 4], and therefore the investigation of IBD pathogenesis has been mainly focused on immune abnormalities.

Free fatty acids (FFAs) are not only essential fuel sources of humans and animals, but also important signaling molecules contributing to many cellular functions through the activation of “metabolite-sensing” G protein-coupled receptors (GPCRs) including GPR40 (FFAR1), GPR41 (FFAR3), GPR43 (FFAR2), GPR84, and GPR120 (FFAR4), which were also called as FFA receptors (FFARs) [5, 6]. FFAs with different chain length regulate functions varying from nutrient sensing, adipogenesis, and GLP-1 secretion to immunoregulation [6–9]. Accumulating lines of evidence in recent years have shown that FFAs might influence intestinal inflammation and diseases [10–13]. These effects are mostly regulated by short-chain fatty acids (SCFAs), including acetate, propionate, and butyrate [14], which are microbial metabolites abundant in the colonic lumen [14, 15]. SCFAs display protective roles in intestinal inflammation and GPR43^−/−^ mice display an increased severity of colitis [10]. Mice with deletion of GPR109A, a GPCR activated by butyrate and niacin, also show an enhanced susceptibility to colitis and colon cancer [11]. In addition, long-chain fatty acids such as docosahexaenoic acid have been observed to ameliorate intestinal mucosal inflammation by activating GPR120 in IL-10^−/−^ mice [13]. However, the roles of medium-chain fatty acids (MCFAs) and their receptors in the induction of intestinal inflammation are still not fully understood.

As a medium-chain fatty acid receptor, GPR84 is a poorly characterized GPCR that couples to pertussis-toxin sensitive Gi signaling [16], which is primarily expressed in ﻿myeloid cells that constitute the innate immune system, including neutrophils, monocytes, and macrophages in the periphery and microglia in the brain [16, 17]. Its expression can be remarkably increased in leukocytes and adipocytes when encountering acute inflammatory stimuli such as lipopolysaccharide (LPS) [16, 18] and tumor necrosis factor-α (TNF-α) [19]. Activation of GPR84 by MCFAs results in increased chemotactic responses [20], enhanced macrophage phagocytosis [21], and promoted secretion of cytokines such as IL-12 p40 [16]. On the contrary, GPR84^−/−^ macrophages exhibit decreased production of pro-inflammatory mediators (IL-6, IL-1β, and TNF-α) [22] upon LPS stimulation, and GPR84^−/−^ T cells produce higher levels of cytokines including IL-4, IL-5 and IL-13 [23]. These studies suggest that the GPR84 signaling may be associated with immune response regulation.

GPR84 is suspected to be involved in several inflammatory and metabolic disorders, including acute lung injury [24], neuropathic pain [22], atherosclerosis [25], reflux esophagitis [26], as well as kidney and pulmonary fibrosis [27, 28]. A GPR84 antagonist, GLPG1205, has been tested in Phase II clinical trial to treat UC [29]. These data indicate that GPR84 may be a target of therapeutic value, and all GPR84 ligands with activating or inhibitory effects may have potential clinical applications. However, the exact roles of GPR84 in inflammatory diseases such as colitis are still largely unclear and needed to be further explored.

Here we sought to study the role of GPR84 in colitis with GPR84^−/−^ mice, a newly discovered GPR84 antagonist CLH536 and colon biopsy samples from UC and CD patients. We demonstrate that the expression of GPR84 is highly correlated with active UC (A-UC) in patients. Genetic deletion or chemical blockade of this receptor shows significant protective effect on dextran sulfate sodium (DSS)-induced colitis model. Further study indicates GPR84 activation imposes pro-inflammatory properties in colonic macrophages, by enhancing NLRP3 inflammasome activation. These results define a unique role of GPR84 in intestinal inflammation, and suggest that GPR84 might be a potential drug target for the treatment of UC.

## Materials and methods

### Antibodies and reagents

DSS Salt (36,000–50,000 M.Wt.) was purchased from MP Biomedicals. LPS, from *Escherichia coli* O111:B4) was purchased from Sigma-Aldrich. Macrophage colony-stimulating factor (M-CSF), interferon-γ (IFN-γ), interleukin (IL)-4, IL-13, transforming growth factor-β1, IL-12, IL-6, IL-2, TNF-α, IL-23, and IL-1β were purchased from R&D Systems. Anti-mouse inducible nitric oxide synthase (iNOS) (6/iNOS/NOS Type II), anti-mouse CD3 (145-2C11), anti-mouse CD28 (37.51), and anti-mouse IFN-γ (R4-6A2) antibodies were purchased from BD Pharmingen. Anti-GPR84 (H-300), anti-F4/80 (BM8), and anti-CD86 (D-6) were purchased from Santa Cruz Biotechnology. Goat anti-rat IgG Alexa Fluor 555, goat anti-mouse IgG Alexa Fluor 555, goat anti-rabbit IgG Alexa Fluor 488, and goat anti-mouse IgG Alexa Fluor 647 antibodies were purchased from Invitrogen. FITC-anti-mouse CD45 (30-F11), PE-Cyanine7-anti-mouse MHCII (M5/114.15.2), APC-anti-mouse Ly-6C (HK1.4), and PE-anti-mouse CD64 (X54-5/7.1) antibodies were purchased from Bioscience. PE-Texas Red-anti-mouse CD11b (M1/70.15) and BB700-anti-mouse Ly-6G(1A8) antibodies were purchased from BD Biosciences. Anti-mouse glyceraldehyde 3-phosphate dehydrogenase (GAPDH) (14C10), anti-mouse NLRP3 (D4D8T), anti-mouse IL-1β (D6D6T), anti-mouse cleaved-IL-1β (E7V2A), and horseradish peroxidase-linked anti-rabbit IgG antibodies were purchased from Cell Signaling Technology.

### Mice

Female C57BL/B6 mice were purchased from the Shanghai Laboratory Animal Center (Shanghai, China). GPR84^−/−^ mice on the C57BL/B6 background were generated at the Shanghai Institute of Materia Medica, Chinese Academy of Sciences (SIMM, CAS, Shanghai, China) using a TALEN system as described in Supplementary Fig. S1. A founder mouse with 13 bp deletion starting from the 69th base of the transcription starting site in exon 2 in one allele, which leads to frameshift and premature translation stop at 23th amino acid, was used to breed GPR84^−/−^ mice. All mice were maintained under specific pathogen-free conditions in the animal facility of the SIMM, with a 12 h light cycle and given a regular chow and water *ad libitum*. Mice were used at 8–10 weeks of age. Animal experiments in this study were approved and conducted in accordance with the guide lines of the Institutional Animal Care and Use Committee at SIMM.

### Patients and tissues

Colonoscopic biopsies were obtained from inflamed and uninflamed sites of the colons from 20 patients with active CD (A-CD), 13 CD patients in remission, 21 patients with A-UC, and 11 UC patients in remission, as well as from normal colonic mucosa of 24 healthy individuals. All patients enrolled in this study were from the Department of Gastroenterology at the Shanghai Tenth People’s Hospital of Tongji University (Shanghai, China). The baseline characteristics were described in Supplementary Table 1. The diagnosis of CD or UC was based on clinical, radiological, and endoscopic examination and histologic findings. The disease severity was assessed according to international standard criteria such as the CD activity index for the diagnosis of CD patients and Mayo scores for UC patients. The study was approved by the Institutional Review Board for Clinical Research of the Shanghai Tenth People’s Hospital of Tongji University. Written informed consent was also obtained from all subjects before the study protocol.

### DSS-induced colitis in mice and drug treatment

The DSS-induced colitis model in mice at age of 8–10 weeks and weighing 20–21 g was established as reported previously [30, 31]. Briefly, for acute colitis, mice were given drinking water with 2.5% DSS for 5 days, followed by DSS-free water. For chronic colitis, mice were given drinking water with 2% DSS for 5 days, followed by 5 days of regular water. This cycle was repeated three times. The severity of colitis was scored daily by recording standard parameters including body weight, stool consistency, and rectal bleeding. The disease activity index (DAI) was measured as reported previously [32, 33]. On day 7, the entire colon and other tissues including spleen, lymph node, and mesenteric lymph node were removed from all killed mice, and the length of the colon was measured and recorded. Each colon sample was divided equally into two parts. One part of the colon samples was fixed in 4% (*w*/*v*) paraformaldehyde, embedded in paraffin, sectioned, and stained with hematoxylin and eosin for histological analysis and immunofluorescence staining; the other part of the colon samples was frozen at −80 °C for quantitative real-time (qRT)-PCR assay, and enzyme-linked immunosorbent assay (ELISA). The histologic grading of colonic inflammation was graded from 0 to 4 as described previously [31]. For drug treatment, mice received vehicle, CLH536 (10 and 30 mg/kg), and sulfasalazine (30 mg/kg), respectively, by oral gavage twice daily from day 0 until the end of the study. Carboxymethylcellulose sodium (0.5%, *w*/*v*) was given as vehicle control.

### AOM/DSS-induced colitis-associated cancer in mice

Colitis-associated cancer (CAC) was induced in mice as reported elsewhere [31, 34]. Briefly, 8- to 10-week-old female GPR84^−/−^ and wild-type (WT) C57BL/B6 mice were first injected intraperitoneally with 10 mg/kg azoxymethane (AOM) (Sigma-Aldrich). Seven days after AOM injection, the mice were given drinking water with 1.5% DSS for 7 days, followed by 14 days of regular water. This cycle was repeated twice (Supplementary Fig. S2a) followed by regular drinking water until the end of experiment. During the study, body weight, stool consistency, and rectal bleeding were monitored and described as DAI. At day 77, all mice were killed and colons were then cut open longitudinally. The presence of tumor nodules was measured and quantified. Tissue samples were also fixed in 4% (*w*/*v*) paraformaldehyde, embedded in paraffin, sectioned, and stained with hematoxylin and eosin or anti-mouse Ki-67 (D3B5, Cell Signaling Technology).

### Isolation of lamina propria mononuclear cells

For epithelial layer stripping, colons were agitated in 5 mM EDTA at 37 °C for 30 min, and then digested in collagenase IV at 37 °C for 2 × 30 min. Following that specimens were enriched with a 40% and 70% Percoll (GE Healthcare) gradient to remove epithelil cells. In some experiments, lamina propria (LP) macrophages were analyzed by flow cytometry. Gating strategy in Supplementary Fig. S4 was based on previous report [35–38]. Briefly, following initial gating on live CD45^+^ cells, CD11b^+^Ly6G^−^ cells were then gated based on SSC and CD64^+^ and finally on Ly6C and MHCII.

### Generation of BMDM

Bone marrow (BM) was flushed from femur and tibia bones. BM cells were cultured in Dulbecco’s modified Eagle’s medium (DMEM) supplemented with 10% fetal bovine serum (FBS), 100 IU/mL penicillin, 100 μg/mL streptomycin, and 25 ng/mL M-CSF, and were allowed to differentiate for 7 days. Media were supplemented every 2–3 days. For generation of M1 macrophages, BM-derived macrophages (BMDMs) were stimulated for 24 h with 100 ng/mL LPS and 20 ng/mL IFN-γ. To generate M2 macrophages, BMDM were cultured for 24 h with 20 ng/mL IL-4, 10 ng/mL IL-13.

### Generation of BM chimeras

BM cells were flushed and collected from the femurs of WT or GPR84^−/−^ mice and transferred via intravenous injection into lethally irradiated (800 rads) WT or GPR84^−/−^ recipients (1 × 10^7^ cells/mouse). Two months after reconstitution, chimeric mice were used for DSS-induced colonic inflammation experiments.

### Isolation of intestinal epithelial cells

Intestinal epithelial cells (IECs) were isolated as described previously [39, 40]. Briefly, the colon was removed from the sacrificed mice, cut into 0.5 cm pieces and placed in cold phosphate-buffered saline to remove debris. Primary IECs were dissociated by incubating at 37 °C for 2 × 20 min in phosphate-buffered saline with 2 mM dithiothreitol and 1 mM EDTA under gently shaking condition. The cells were then collected and further purified via density gradient centrifugation with 20% and 40% percoll-RPMI solution.

### Reverse-transcription and real-time PCR

Total RNA was extracted from mouse tissues or human biopsies using TRIzol (Invitrogen). The RNA was subjected to reverse transcription with random hexamer primer and Moloney murine leukemia virus reverse transcriptase (Promega). Real-time PCR was conducted in the LightCycler quantitative PCR apparatus (Stratagene) using SYBRGreen PCR kit (TaKaRa). Expression value was normalized to GAPDH in the same sample and then normalized to the control. The sequences of the primer pairs are provided in Supplementary Table 2.

### Inflammasome activation and inhibition

Murine BMDMs were stimulated with LPS (100 ng/mL) for 3 h in DMEM with 10% FBS, followed by 30 min stimulation with nigericin (10 μM) or ATP (5 mM) and 6-OAU (30 μM or as indicated) in Opti-MEM medium (Life Technologies), or treatment with aluminum hydroxide (Alum, 150 μg/mL) or monosodium urate crystal (MSU, 500 μg/ml), and 6-OAU (30 μM) for 4 h. In the experiments with an antagonist of GPR84, CLH536, BMDMs were cultured under stimulation with LPS and CLH536 was added 30 min prior to the treatment with nigericin (10 μM) and 6-OAU (30 μM). Supernatants, lysates, and cell pellets were collected for the following ELISA and Western blot analyses, respectively.

### Macrophage-epithelial cell coculture and transepithelial electrical resistance (TER)

Caco-2 cells were seeded at 4 × 10^5^ cells/well on 24-well Transwell inserts (0.4 μm pore size, Corning) in DMEM supplemented with 10% FBS, 1% non-essential amino acids, 2% glutamine, 100 U/mL of penicillin, and 100 μg/mL of streptomycin. Cell culture medium was changed every 3 days. Caco-2 cells that had been cultured for 10 days and formed an integrated cell monolayer with a TER of more than 150 Ω·cm^2^ were used for this study. BMDMs from WT or GPR84^−/−^ mice pre-seeded onto the 24-well plate were stimulated with 100 ng/mL LPS and 20 ng/mL IFN-γ for 24 h to generate M1 macrophages. After replacing all media with DMEM, the Transwell inserts on which the Caco-2 cells had been cultured were added into the wells pre-seeded with M1 macrophages. Forty-eight hours later, the damage to the Caco-2 monolayers was evaluated by TER measurement with a Millicell-ERS instrument (Millipore, Bedford, MA, USA).

### Calcium mobilization assay

HEK293 cells stably expressing Gα16 and various GPCRs were seeded onto 96-well plates and incubated for 24 h. Cells were loaded with 2 μM Fluo-4 AM in HBSS at 37 °C for 45 min. After removal of excess dye, 50 μL HBSS containing antagonists was added. After incubation at room temperature for 10 min, 25 μL agonist of the receptor was dispensed into the wells using a FlexStation III microplate reader (Molecular Devices) and intracellular calcium change was recorded at an excitation wavelength of 485 nm and an emission wavelength of 525 nm.

### Statistical analysis

Data were analyzed with GraphPad Prism software (GraphPad Software). Nonlinear regression analyses were performed to generate dose–response curves and calculate EC_50_ or IC_50_ values. Data are presented as means ± SEM. Two-way analysis of variance test was used to assess the significance between treatment groups of colitis animals. For any given date, the DAI scores were analyzed using a non-parametric Mann–Whitney *U*-test. For other analyses, including gene expression, cytokine secretion, cell percentage, and histological analysis, the significance was assessed by two-tailed Student’s *t*-test. The *P*-values < 0.05 were considered statistically significant. Pearson’s correlation was performed to analyze the correlation of the mRNA levels of GPR84 in colonic mucosa and Mayo score of UC patients.

## Results

### GPR84 deficiency ameliorates DSS-induced colitis in mice

**Fig. 1:**
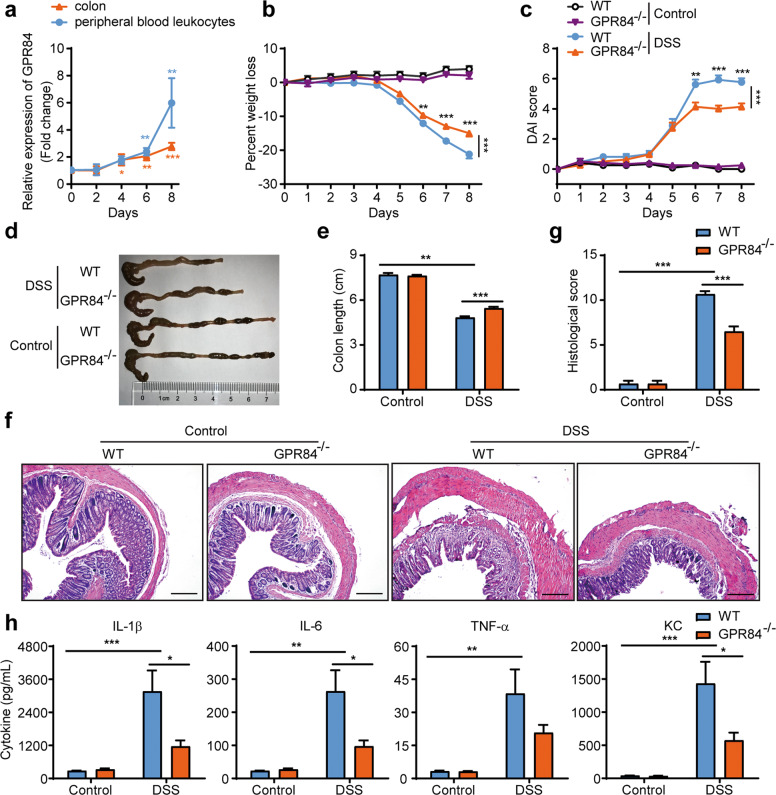
GPR84 deletion alleviates clinical symptoms of DSS-induced colitis in mice. **a** Upregulation of GPR84 mRNA in colon and peripheral blood leukocytes during DSS-induced colitis in mice. mRNA was isolated from colon and peripheral blood leukocytes of DSS-treated mice (*n* = 12) on days 0, 2, 4, 6, 8. Gene expression was normalized to GAPDH in the same sample and then normalized to day 0. *P < 0.05, **P < 0.01, ***P < 0.001 vs. day 0. **b**, **c** Change of body weight (**b**) and disease activity index (DAI) (**c**) during DSS-induced colitis in mice (*n* = 12–28 mice/group). **P < 0.01, ***P < 0.001 vs. DSS-treated WT mice at the same time points (two-way ANOVA with Mann–Whitney *U*-test). **d**, **e** Representative photograph (**d**) and statistical analysis of colon length (**e**) at day 7 in DSS or vehicle-treated mice (*n* = 12), **P < 0.01, ***P < 0.001. **f**, **g** Representative images of H&E-stained colon sections (**f**) and statistical analysis of histological scores (**g**) at day 7 after DSS induction (*n* = 12), Scale bars = 200 μm, ***P < 0.001. **h** Cytokine production in the colon at day 7 after DSS-induction (*n* = 12), *P < 0.05, **P < 0.01, ***P < 0.001. All data are presented as means ± SEM.

To investigate whether GPR84 is involved in the pathogenesis of colitis, we first checked the expression level of GPR84 on days 0, 2, 4, 6, and 8 in DSS-induced mouse model of acute colitis. In both colon and peripheral blood leukocytes (Fig. 1a), GPR84 was significantly upregulated starting on day 4 and peaked on day 8, as compared to that on day 0, suggesting that increased GPR84 expression was associated with colitis. GPR84 knockout (GPR84^−/−^) mice were generated with TALEN system [41–43] (Supplementary Fig. S1a, b) and colitis was induced with DSS. Compared with WT mice, GPR84^−/−^ mice developed significantly less severe colitis with reduced body weight loss and DAI (Fig. 1b, c). Decreased disease severity was also accompanied by a reduction of colon shortening (Fig. 1d, e). The colon histopathology revealed that GPR84^−/−^ mice had less inflammatory cell infiltration and mucosal damage, including epithelial ulceration, crypt loss, and submucosal edema (Fig. 1f, g). Consistent with the histopathology in the colon, the proinflammatory cytokines (TNF-α, IL-1β, and IL-6) and keratinocyte-derived cytokine (KC) were also significantly reduced in colon tissues from GPR84^−/−^ mice (Fig. 1h).

Chronic inflammation has been suggested to contribute to the development and progression of cancers. In patients with IBD, such as UC, the risk of CAC development is much higher than in the general population [44]. To further explore function of GPR84 in chronic intestinal inflammation and carcinogenesis, a model of inflammation-associated colon cancer was induced with intraperitoneal injection of AOM, followed by cyclic DSS (1.5%) treatment (Supplementary Fig. S2a), as described previously [31, 34]. Over the duration of inflammation and carcinogenesis induction, GPR84^−/−^ mice displayed less weight loss, compared with the WT mice (Supplementary Fig. S2a). At the end of the AOM + DSS treatment regime, a trend of reduced number of polyps in the colon could be observed in GPR84^−/−^ mice, especially polyps in small size (<2 mm, *P* = 0.0716) (Supplementary Fig. S2b–d). Analysis of Ki-67 expression revealed no significant difference in cell proliferation in colon between WT and GPR84^−/−^ mice, before or after AOM + DSS treatment (Supplementary Fig. S2e, g). However, histopathological analysis of the colon tissue confirmed that GPR84^−/−^ mice had significantly less inflammatory cell infiltration and mucosal damage (Supplementary Fig. S2e, f). These observations suggest GPR84 deficiency may reduce the formation of initial polyps, but once tumor forms, GPR84 does not affect the growth, suggesting that GPR84 largely participates in inflammation regulation.

### GPR84 antagonist attenuates the severity of DSS-induced acute colitis

**Fig. 2:**
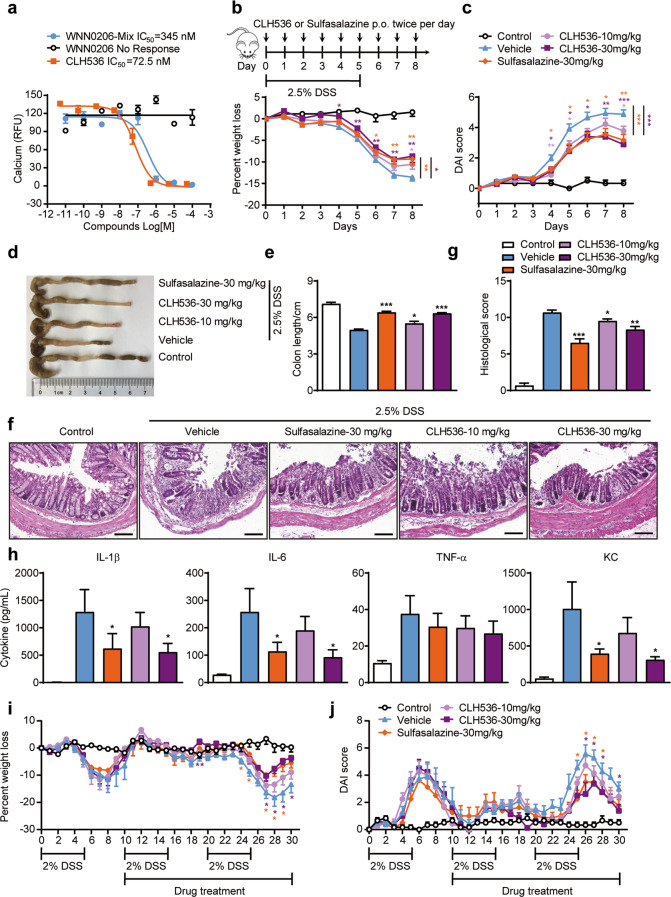
GPR84 receptor antagonist alleviates pathogenesis of DSS-induced colitis in mice. **a** Dose–response of CLH536, WNN0206-Mix, and WNN0206 in inhibiting 6-OAU-induced calcium responses in HEK293/Gα16/GPR84 cells. **b**, **c** Change in the body weight (**b**) and DAI score (**c**) of DSS-treated mice receiving vehicle, sulfasalazine (30 mg/kg) and CLH536 (10 or 30 mg/kg) by oral administration (*n* = 18), *P < 0.05, **P < 0.01, ***P < 0.001 (two-way ANOVA with Mann–Whitney *U*-test) vs. DSS-induced mice receiving vehicle. **d**, **e** Representative photographs (**d**) and statistical analysis of the length (**e**) of colons at day 7 after treatment. *P < 0.05, ***P < 0.001 vs. DSS-induced mice receiving vehicle. **f**, **g** Representative images of H&E-stained colon sections (**f**) and statistical analysis of histological scores (**g**) at day 7 after various treatments (*n* = 12). Scale bars = 50 μm, *P < 0.05, **P < 0.01, ***P < 0.001 vs. DSS-induced mice receiving vehicle. **h** Cytokine production in the colon at day 7 after various treatments (*n* = 12), *P < 0.05 vs. DSS-induced mice receiving vehicle. **i**, **j** Change in the body weight (**i**) and DAI score (**j**) in mice with DSS-induced chronic colitis receiving vehicle, sulfasalazine (30 mg/kg) and CLH536 (10 or 30 mg/kg) by oral administration started on Day 10 (*n* = 8), *P < 0.05, **P < 0.01 (two-way ANOVA with Mann–Whitney *U*-test) vs. DSS-induced mice receiving vehicle. All data are expressed as means ± SEM.

As the knockout of GPR84 significantly alleviated the clinical symptoms of colitis in mice, we wondered whether a receptor antagonist may have similar effects. Our lab has been working on the discovery of GPR84 ligands since 2014 [45, 46]. In a high-throughput screening of 160,000 compounds using a calcium mobilization assay with HEK293 cells expressing GPR84, we not only identified 2-(hexylthio) pyrimidine-4,6-diol (ZQ-16) as a novel agonist with an EC_50_ of 134 nM [45], but also a novel antagonist CLH536, which originally came from an impurity in sample WNN0206 (Fig. 2a and Supplementary Fig. S3a). The original sample WNN0206 with active impurity showed IC_50_ of 345 nM, whereas the pure WNN0206 sample synthesized according to the reported procedure [47] had no antagonist activity on GPR84. The active impurity CLH536 was then isolated and identified from the original sample as a novel diaryl phosphodiester scaffold, which is a by-product in the two-step synthesis of WNN0206, from the dimerization of the unconsumed ketone D1 after step 1 (Supplementary Fig. S3b).

CLH536 blocked 6-OAU (1 μM) induced GPR84 activation with an IC_50_ of 72.5 nM (Fig. 2a), while displayed no agonist or antagonist functions on other FFARs (GPR40, GPR41, GPR119, and GPR120) and a number of other GPCRs at concentrations up to 10 μM (Supplementary Table 3), indicating excellent selectivity for GPR84. CLH536 was then tested in DSS-induced colitis. Sulfasalazine, a currently marketed anti-IBD drug, was used as a positive control. Compared to vehicle, both CLH536 and sulfasalazine administered at 30 mg/kg reduced the loss of body weight (Fig. 2b). The DAI was also significantly decreased with CLH536 and sulfasalazine treatment (Fig. 2c). CLH536 also reduced colonic shortening in both dosages (Fig. 2d, e). Histopathological analysis of the colon tissue revealed that CLH536 significantly reduced inflammatory cell infiltration and mucosal damage (Fig. 2f, g). Proinflammatory cytokines (TNF-α, IL-1β, and IL-6) and KC were also markedly reduced in colon tissues (Fig. 2h) by CLH536 treatment.

IBD are clinically characterized as chronic, relapsing, and remitting disorders. The therapeutic effect of CLH536 was also evaluated in DSS-induced chronic colitis after the first induction cycle. Compared to vehicle, both CLH536 and sulfasalazine administered at 30 mg/kg significantly reduced the loss of body weight and DAI score (Fig. 2i, j). Together, these data demonstrate that blocking GPR84 with a small molecule antagonist protects against the intestinal mucosal inflammation in DSS-induced colitis, further confirming the involvement of GPR84 in IBD.

### GPR84 signaling in hematopoietic cells plays a critical role in intestinal inflammation

**Fig. 3:**
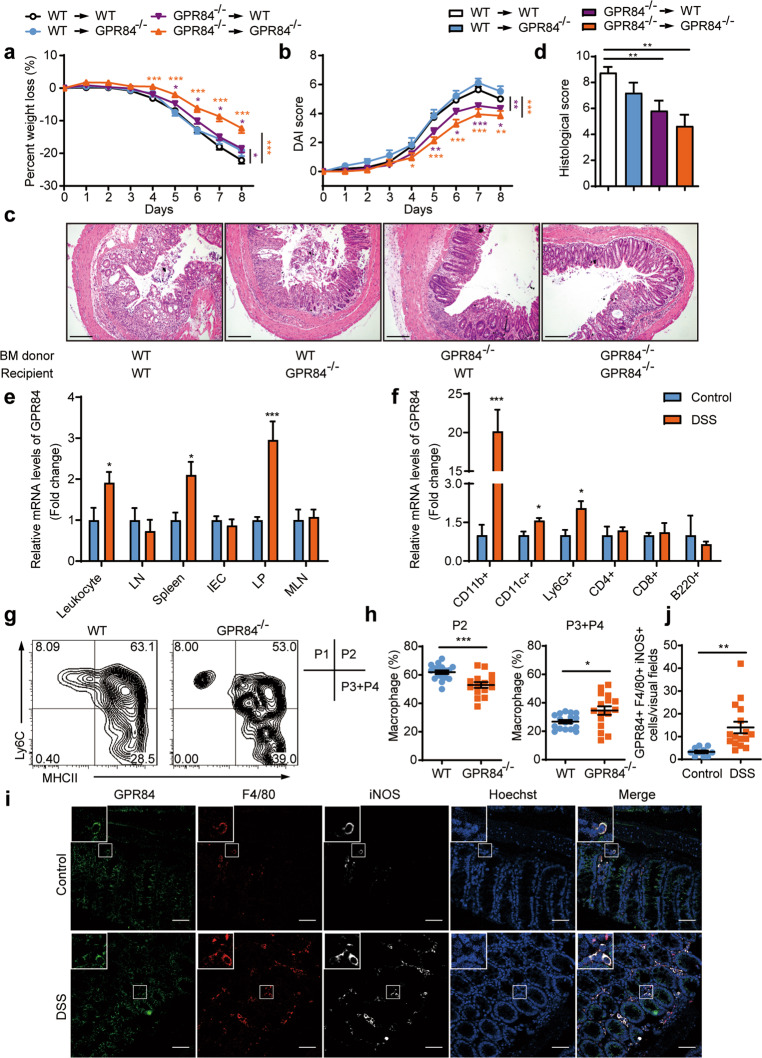
GPR84 deletion leads to the reduction of the proinflammatory intestinal macrophages. **a**, **b** Bone marrow chimeras were constructed by transplanting bone marrow cells isolated from WT or GPR84^−/−^ donor mice into lethally irradiated WT or GPR84^−/−^ recipient mice via i.v. route (*n* = 7–33 mice/group). Two months after reconstitution, mice were challenged with DSS for 5 consecutive days. Changes of body weight (**a**) and DAI (**b**) were recorded. *P < 0.05, **P < 0.01, ***P < 0.001 vs. DSS-treated WT–WT chimeric mice (two-way ANOVA with Mann–Whitney *U*-test). **c**, **d** Representative images of H&E-stained colon sections (**c**) and statistical analysis of histological scores (**d**) at day 7. Scale bars = 200 μm, **P < 0.01. **e**, **f** qRT-PCR analysis of GPR84 expression in various tissues (**e**) and colonic lamina propria cells (**f**) of control and DSS-induced mice on day 7. Gene expression was normalized to GAPDH in the same sample and then normalized to control. LN, lymph node; IEC, intestinal epithelial cells; LP, colonic lamina propria; MLN, mesenteric lymph node. *P < 0.05, ***P < 0.001 vs. control (*n* = 6 mice/group). **g**, **h** Representative flow cytometry plots of macrophage subsets (**g**) in colonic lamina propria of DSS-treated WT and GPR84^−/−^ mice on day 7, followed by quantification of the pro- and anti-inflammatory populations (**h**). Proinflammatory population was defined as Ly6C^+^MHCII^+^ cells and anti-inflammatory as Ly6C^−^MHCII^+^. *P < 0.05, ***P < 0.001. **i**, **j** Representative pictures (**i**) and statistical analysis (**j**) of immunofluorescence staining of GPR84^+^F4/80^+^iNOS^+^ proinflammatory macrophages in colon sections from control and DSS-induced mice on day 7. Nuclei were stained with Hoechst 33258 (blue). Scale bars = 50 μm. **P < 0.01, *n* = 10–16 mice/group, 3–6 visual fields were counted for each mouse. All data are presented as means ± SEM.

To further determine whether GPR84 plays a role in haematopoietic cells such as immune cells or non-hematopoietic cells such as intestinal cells in the development of colitis, BM chimeras were generated by transferring WT or GPR84^−^^/−^ BM cells to lethally irradiated WT or GPR84^−/−^ mice. The chimeras were then subjected to DSS treatment as described above. GPR84 in hematopoietic cells appeared to play a critical role in DSS-induced colonic inflammation because both the WT and GPR84^−/−^ hosts that received GPR84^−/−^ BM developed a mild colitis with a significantly reduced body weight loss, lower DAI score and less mucosal damage compared with corresponding hosts receiving WT BM cells (Fig. 3a–d). And the non-haematopoietic GPR84 showed little effect on colitis because the WT and GPR84^−/−^ hosts received WT BM exhibited similar level of colitis with almost same body weight loss, DAI score and mucosal damage (Fig. 3a–d). Therefore, these data highly suggest that GPR84 may participate in intestinal inflammation by regulating hematopoietic cell (most likely the immune cell) functions.

### GPR84 regulates colitis by mediating the composition of the intestinal macrophage pool

Detailed expression analysis with quantitative reverse-transcription PCR revealed that GPR84 mRNAs were significantly upregulated in immune cells/tissues including peripheral blood leukocytes, spleen, and especially colonic LP at day 7 in DSS-induced colitis (Fig. 3e). The immune cells were then isolated from colonic LP in DSS-treated and control mice. GPR84 was found to be most significantly upregulated in CD11b^+^ macrophages (day 7) (Fig. 3f), strongly suggesting a role of GPR84 in macrophages during colitis. In addition, GPR84 was also upregulated to a less extent in both the Ly6G^+^ neutrophils and CD11c^+^ dendritic cells after DSS treatment (Fig. 3f). We next sought to investigate whether GPR84 regulates macrophage population in colitis. We followed the nomenclature for subsets of intestinal macrophage in previous publications [35–37], showing that circulating monocytes migrate into the LP and undergo a four-stage process of differentiation, including the proinflammatory P2 (Ly6C^+^MHCII^+^) stage and the anti-inflammatory P3 and P4 (Ly6C^−^MHCII^+^) stages (Supplementary Fig. S4). LP cells were isolated from GPR84^−/−^ and WT mice at day 7 after DSS treatment. Flow cytometry analysis revealed that the proinflammatory P2 macrophages were significantly reduced in the colitic GPR84^−/−^ mice accompanied by a concomitant increase in anti-inflammatory P3 and P4 macrophages (Fig. 3g, h). Further analysis revealed that GPR84^−/−^ also led to slight decrease of neutrophils in the LP, but did not affect the DC population (Supplementary Fig. S5a–d). Immunofluorescence staining of colon sections from WT mice demonstrated that the iNOS^+^ pro-inflammatory macrophages expressed high level of GPR84 and were significantly increased in colonic mucosa after DSS treatment (Fig. 3i, j). Collectively, these data suggest an important role of GPR84-dependent signals in the differentiation of intestinal macrophages during mucosal inflammation.

### GPR84 mediates the pathogenesis of UC in patients

**Fig. 4:**
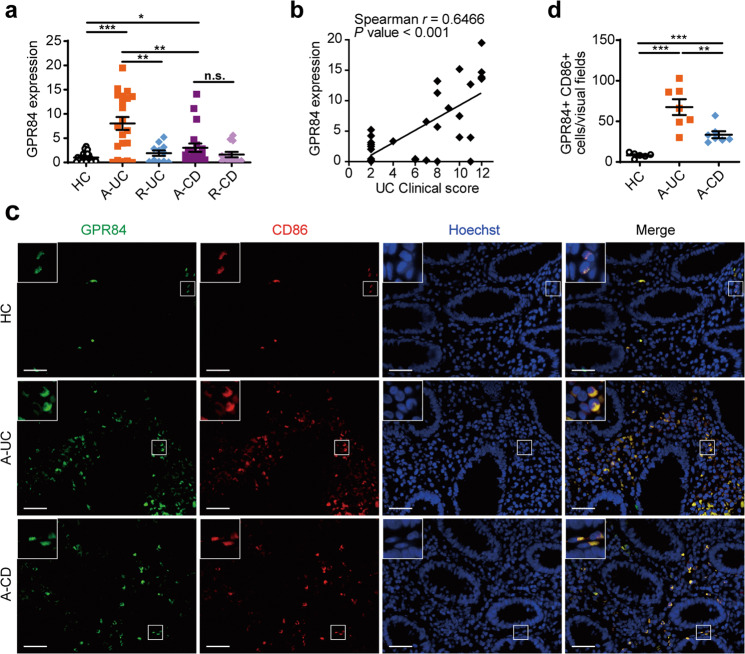
GPR84 is highly correlated with IBD in human, especially active UC patients. **a** qRT-PCR analysis of GPR84 expression in colonic biopsies from healthy controls (HC, *n* = 24) and inflamed mucosa from patients with active ulcerative colitis (A-UC, *n* = 21), remissive UC (R-UC, *n* = 11), active Crohn’s disease (A-CD, *n* = 20), or remissive CD (R-CD, *n* = 13). **b** Correlation between the mRNA levels of GPR84 in the colon sample and Mayo clinical score of the UC patients (*n* = 32). Pearson’s rank correlation coefficient *r* = 0.6466, *P* < 0.001. **c** Representative pictures of immunofluorescence staining for GPR84^+^CD86^+^ proinflammatory macrophages in colon sections from healthy controls (HC) and patients with active UC or CD. Nuclei were stained with Hoechst 33258 (blue). GPR84 (green), CD86 (red), and merged image are shown. Scale bars = 40 μm. **d** Quantification of GPR84^+^CD86^+^ proinflammatory macrophages in **c**. For each individual, 3~6 different visual fields were counted. *n* = 6 for HC, *n* = 7 for A-UC, and *n* = 8 for A-CD. Each symbol represents an individual subject; horizontal lines indicate means ± SEM; *P < 0.05, **P < 0.01, ***P < 0.001.

As GPR84 mediates the development of colitis in mice, we wondered whether GPR84 plays a similar role in IBD patients. Colonic biopsies were collected from IBD patients, including both CD and UC patients in their active or remissive stages. The GPR84 mRNA levels were found to be most significantly upregulated in the colonic samples from A-UC patients and to a lesser extent in the A-CD patients, but not in either types of IBD patients in their remissive stage (Fig. 4a). Most interestingly, the expression of GPR84 in the colon samples of UC patients was positively correlated with Mayo clinical score (Spearman’s rank correlation coefficient *r* = 0.6466, *P* < 0.001) (Fig. 4b), indicating that GPR84 may play a negative role in UC patients. As GPR84 was highly upregulated in macrophages and affected macrophage polarization in the LP of the colitis mice, we performed double immunofluorescence staining for GPR84 and CD86 in colonic mucosa sections from patients and healthy controls. The vast majority of infiltrating CD86^+^ macrophages were found to also express GPR84 and the number of the CD86^+^GPR84^+^ cells was significantly increased in the colonic mucosa of patients with A-UC or CD, especially patients with UC, compared with healthy controls (Fig. 4c, d). Taken together, these data indicate that GPR84 signaling may participate in the pathogenesis of IBD, especially UC, in human patients, and macrophages might be the major type of cells regulated by GPR84.

### GPR84 regulates proinflammatory macrophage polarization in vitro

**Fig. 5:**
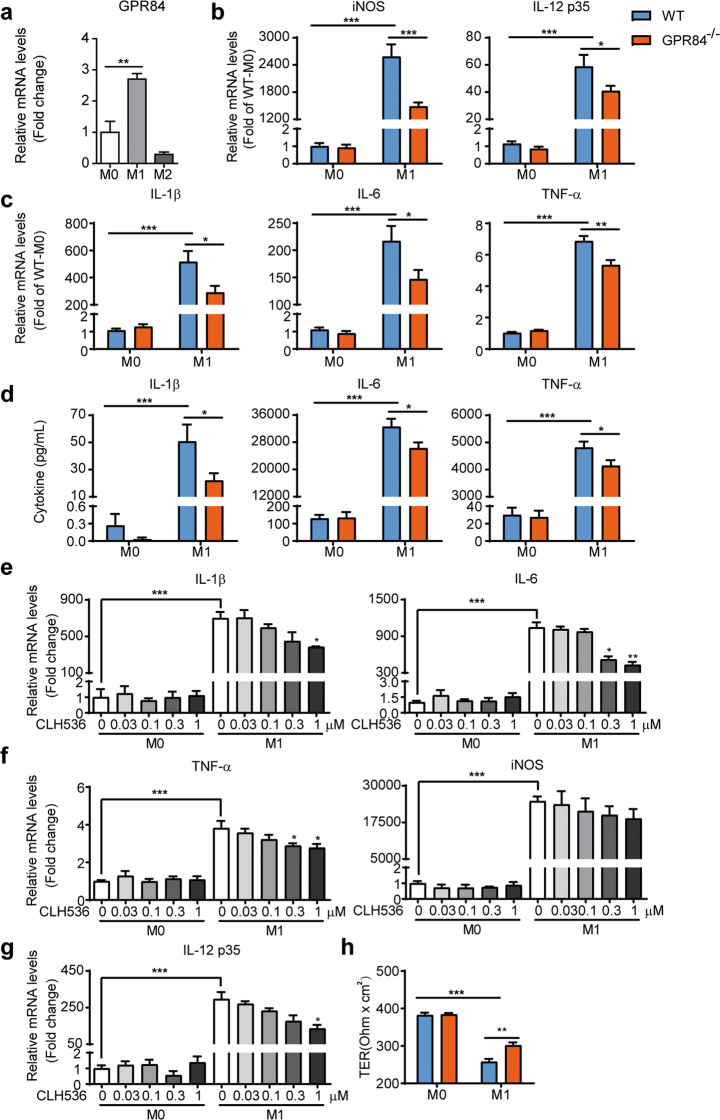
GPR84 knockout or blockade reduces the proinflammatory M1 polarization of BMDM. **a** qRT-PCR analysis of GPR84 expression in unpolarized (M0), classically activated (M1), or alternatively activated (M2) BMDMs. **P < 0.01 (*n* = 4). **b**, **c** qRT-PCR analysis of iNOS and pro-inflammatory cytokine expression in BMDM cultured in M0 or M1 conditions for 24 h. Results were normalized to GAPDH expression in the same sample and then normalized to the control (WT M0 BMDM). *P < 0.05, **P < 0.01, ***P < 0.001 (*n* = 4). **d** ELISA analysis of cytokines in the supernatants of BMDM cultured in M0 or M1 conditions for 24 h. *P < 0.05, ***P < 0.001 (*n* = 4). **e**–**g** qRT-PCR analysis of iNOS and pro-inflammatory cytokine expression in BMDM cultured in M0 or M1 conditions in the presence of various concentrations of CLH536 for 24 h. Results were normalized to GAPDH expression in the same sample and then normalized to the control (WT M0 BMDM). *P < 0.05, **P < 0.01, ***P < 0.001 vs. M1 condition without CLH536 (*n* = 6). **h** TER of the epithelial cell (Caco-2) layer measured after 48 h coculture with WT or GPR84^−/−^ M1 macrophages. **P < 0.01, ***P < 0.001 (*n* = 6). All data are expressed as means ± SEM.

Given that GPR84 may regulate colonic macrophage differentiation during colitis, we then studied whether GPR84 could affect macrophage polarization in vitro. To this end, WT BMDMs and peritoneal macrophages (PMs) were polarized into pro-inflammatory M1 state with LPS and IFN-γ or anti-inflammatory M2 state with IL-4 and IL-13 in vitro. GPR84 was found to be upregulated in M1 but not M2 state in both BMDMs and PMs (Fig. 5a and Supplementary Fig. S6a). Compared to the WT macrophages, GPR84^−/−^ macrophages expressed significantly less M1-related genes, including *iNOS*, *TNF-α*, *IL-1β*, *IL-6*, and *IL-12 p35*, which was induced by LPS and IFN-γ (Fig. 5b, c and Supplementary Fig. S6b, c). And GPR84^−/−^ M1 macrophages also produced less pro-inflammatory cytokines such as TNF-α, IL-1β, and IL-6 (Fig. 5d and Supplementary Fig. S6d). Similarly, GPR84 antagonist CLH536 also reduced the expression of pro-inflammatory genes associated with M1 (Fig. 5e–g). In contrast, expression of anti-inflammatory cytokines and costimulatory molecules were not significantly changed in GPR84^−/−^ BMDMs cultured in M2 conditions (Supplementary Fig. S7). Together, these data reveal that GPR84 deficiency leads to reduced pro-inflammatory M1 polarization of macrophages in vitro.

To assess the effects of macrophages on the integrity of the intestinal epithelial barrier under inflammatory conditions, we established a gut inflammation model in vitro as described previously [48, 49]. WT and GPR84^−/−^ BMDMs were stimulated with LPS and IFN-γ for 24 h and then co-cultured with intestinal epithelial monolayers formed by Caco-2 cells for 2 days. TER of the monolayers that reflects the integrity of the IECs was monitored. The TER value of Caco-2 cells showed less decrease after co-culturing with GPR84^−/−^ M1 BMDMs, compared to the WT M1 cells (Fig. 5h), suggesting that GPR84 deficiency reduces macrophage-induced intestinal epithelial damage.

### GPR84 regulates pro-inflammatory function of macrophages via enhancing NLRP3 inflammasome activation

**Fig. 6:**
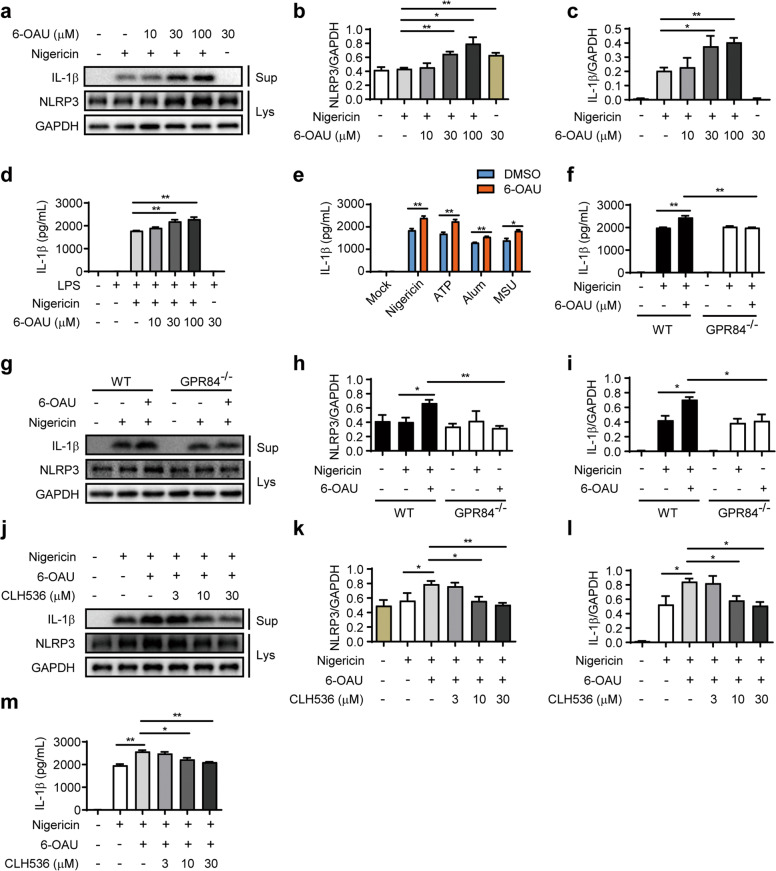
GPR84 regulates NLRP3 inflammasome activation. **a** Representative Western blot analysis of IL-1β in the culture supernatants (Sup) and NLRP3 in the lysate (Lys) of BMDMs primed with LPS for 3 h, and then stimulated with nigericin (10 μM) with various concentrations of 6-OAU for 30 min. **b**, **c** Quantification of NLRP3 (**b**) and IL-1β (**c**) in Western blotting in **a**. Proteins were normalized to GAPDH in the same sample. **d** ELISA analysis of IL-1β in the supernatants of BMDMs primed with LPS for 3 h, and then stimulated with nigericin (10 μM) with various concentrations of 6-OAU for 30 min. **e** ELISA analysis of IL-1β in supernatants from LPS-primed (3 h) BMDMs stimulated with nigericin (10 μM) and ATP (5 mM) for 30 min, or Alum (aluminum hydroxide, 150 μg/mL), and MSU (monosodium urate crystal, 500 μg/mL) for 4 h with or without 6-OAU (30 μM). **f** ELISA analysis of IL-1β in culture supernatants of LPS-primed (3 h) WT or GPR84^−/−^ BMDMs stimulated with nigericin (10 μM) and 6-OAU (30 μM). **g** Representative Western blot analysis of IL-1β in culture supernatants (Sup) and NLRP3 in the lysate (Lys) of LPS-primed (3 h) WT or GPR84^−/−^ BMDMs stimulated with nigericin (10 μM) and 6-OAU (30 μM). **h**, **i** Quantification of NLRP3 (**h**) and IL-1β (**i**) in Western blotting in **g**. Proteins were normalized to GAPDH in the same sample. **j** Representative Western blot analysis of IL-1β in culture supernatants (Sup) and NLRP3 in the lysate (Lys) of LPS-primed BMDMs stimulated with nigericin (10 μM) and 6-OAU (30 μM) for 30 min in the presence of various doses of CLH536. **k**, **l** Quantification of NLRP3 (**k**) and IL-1β (**l**) in Western blotting in (**j**). Proteins were normalized to GAPDH in the same sample. **m** ELISA test of IL-1β in supernatants from LPS-primed BMDMs stimulated with nigericin (10 μM) and 6-OAU (30 μM) for 30 min in the presence of various doses of CLH536. All data are means ± SEM, *P < 0.05, **P < 0.01.

Inflammasome is a group of protein complexes composed of a cytosolic receptor of the Nod-like receptor (NLR) family, an adaptor protein termed ASC (apoptosis-associated Speck-like protein containing an N-terminal caspase recruitment domain CARD), and procaspase-1 [50, 51]. The NLR family member NLRP3 inflammasome is rapidly emerging as a crucial regulator of intestinal homeostasis [52]. This innate immune receptor mediates the assembly of the inflammasome complex in the presence of pathogens and endogenous danger signals [53], triggering activation of caspase-1 and secretion of IL-1β and IL-18, and plays a key role in the pathogenesis of inflammatory diseases including IBD [52, 54, 55]. Our data showed that knockout or blockade of GPR84 significantly reduced IL-1β level in both colitis mice and M1 macrophages, suggesting the possible involvement of NLRP3 inflammasome. To determine whether GPR84 regulates inflammasome activation, LPS-primed BMDMs were challenged with NLRP3 agonist nigericin and GPR84 agonist 6-OAU, and the activation of inflammasome was accessed by the production of IL-1β. 6-OAU alone did not induce IL-1β secretion, however, nigericin-stimulated NLRP3 activation and IL-1β secretion was significantly increased by 6-OAU in a dose-dependent manner (Fig. 6a–d). GPR84 agonist 6-OAU could also enhance the IL-1β production induced by other NLRP3 agonists, including ATP, MSU, and Alum (Fig. 6e). In GPR84^−/−^ BMDMs, nigericin was still able to stimulate IL-1β production, but the enhancement effect of 6-OAU was lost (Fig. 6f–i), suggesting the effect of 6-OAU is indeed mediated via GPR84 activation. In addition, the amplification of nigericin-induced IL-1β production in BMDMs by 6-OAU was also inhibited by GPR84 antagonist CLH536 in a dose-dependent manner (Fig. 6j–m). These results indicate that activation of GPR84 leads to amplified NLRP3 inflammasome activation.

## Discussion

Our data show that GPR84 signaling may participate in the pathogenesis of IBD, especially UC by regulating polarization of macrophages. Macrophages are increasingly recognized as important sentinels in the intestinal immune system and play important roles in chronic intestinal inflammation. In both CD and UC, CD14^hi^ monocytes/macrophages alter markedly in the gut and produce pro-inflammatory cytokines and chemokines, including IL-1β, TNF-α, IL-6, IL-12, and CCL11 [56, 57], responding in an aberrant manner to commensal microbes [56]. And the high levels of TREM1 expressed by these cells can potently amplify pro-inflammatory responses [58]. Similar to human patients, murine colitis models show intense accumulation of Ly6C^hi^ monocytes, which also produce high levels of IL-1β, TNF-α, IL-6, IL-12, and express high level of TREM1 and respond in a highly pro-inflammatory manner to TLR stimulation [59].

Selective deletion of IL-1β or neutralization of TNF-α in Ly6C^hi^ monocytes suppresses colitis in mice [60, 61]. Reducing the recruitment of monocyte to the inflamed mucosa in mice by blockade of CCL2-CCR2 axis also protects mice from chemical-induced colitis [59]. Importantly, the CCL2-CCR2 axis may also govern monocyte recruitment in the gut of human and play important roles in the development of IBD [62]. In addition to their direct effects, monocytes/macrophages can also recruit and support other innate and adaptive immune effector cells, which are important in pathology of IBD. For instance, CD14^hi^ monocytes/macrophages in the IBD mucosa support pathogenic T cell function through IL-23 production [56, 63].

Plasticity and functional polarization are hallmarks of macrophages and they can differentiate into classically activated M1 or alternatively activated M2 subtypes according to environmental cues [64]. M1 macrophages exert pro-inflammatory and anti-microbial activities, while M2 macrophages exhibit anti-inflammatory properties and play important roles in wound healing and fibrosis [65]. The imbalance between M1 and M2 switching is the key point that initiates various disorders. In IBD, the population of M1 like macrophages increases with the reduction of M2 like population, indicating the importance of M1 cells in IBD development [37]. Interventions that reducing the M1 polarization, or enhancing M2 population, or promoting the M1 to M2 conversion has been proposed to treat IBD [59, 66, 67]. Our study revealed that GPR84 activation imposes pro-inflammatory properties in colonic macrophages through enhancing NLRP3 inflammasome activation, while the loss or blockade of GPR84 prevents the M1 polarization and properties of proinflammatory macrophages.

The NLRP3 inflammasome recruits and activates caspase-1 through ASC protein, leading to the process and release of the proinflammatory cytokines IL-1β and IL-18, and modulate the functions of macrophages. NLRP3 inflammasome is typically activated via a two-step manner [68]. The priming signal is generally from microbial components or endogenous cytokines such as TNF-α and IL-β, leading to the activation of NF-κB and transcription of NLRP3 and pro-IL-1β. The activation signals include a variety of stimuli such as RNA viruses, pore-forming toxins, extracellular ATP, and particulate matters such as silica and β-amyloid. Many intracellular events, including ionic flux, mitochondrial dysfunction and reactive oxygen species generation, have been shown to modulate NLRP3 inflammasome activation [68].

Several GPCRs, such as CASR (calcium-sensing receptor) and DRD1 (dopamine receptor D1), have been reported to regulate NLRP3 inflammasome activations [69, 70]. CASR activates the NLRP3 inflammasome by increasing intracellular Ca^2+^ and decreasing intracellular cAMP. In the contrary, DRD1 signaling inhibits NLRP3 inflammasome by increasing intracellular cAMP, which binds to NLRP3 and promotes its ubiquitination and degradation via the E3 ubiquitinligase MARCH7 [69]. These previous studies suggest that cAMP restrains function of NLRP3 inflammasome by inhibiting its assembly. GPR84 is a Gi-coupled receptor that its activation leads to reduced cAMP production by inhibiting adenylate cyclase. So GPR84 signaling possibly augments NLRP3 inflammasome activation by impairing cAMP-induced NLRP3 polyubiquitination and degradation in macrophages.

Consistent with our results, GPR84 has been proposed to function as a pro-inflammatory GPCR, which may play a role in reflux esophagitis [26], IBD [29], neuropathic pain [22], fibrosis [27, 71], and acute myeloid leukemia [72]. Thus, its antagonists may provide therapeutic effect in those diseases. Galapagos NV has reported a GPR84 antagonist GLPG1205 [29], which has been tested in a Phase II clinical trial to treat UC but failed to meet the primary efficacy end point, but no explanations or detailed preclinical studies have been disclosed. PBI-4050, another GPR84 antagonist developed by Liminal BioSciences, which is also a weak GPR40 agonist, significantly attenuates fibrosis in the kidney and liver [28] in animal models. Liminal BioSciences has developed a second GPR84 antagonist, PBI-4547, which is also a dual agonist of GPR40 and GPR120. PBI-4547 has been shown to relieve high-fat diet-induced obesity in mice [73]. CLH536, the GPR84 antagonist reported in this study, does not activate or block other FFARs and displays higher selectivity towards GPR84.

In summary, we demonstrate that the expression of GPR84 is highly correlated with A-UC in patients. Genetic deletion or chemical blockade of this receptor shows significant protective effect on DSS-induced colitis mice. Further study indicates GPR84 activation imposes pro-inflammatory properties in colonic macrophages, by enhancing NLRP3 inflammasome activation. These results define a unique role of GPR84 in intestinal inflammation, and suggest that GPR84 might be a potential drug target for the treatment of UC.

## Supplementary information

Supplementary information
