# Supplementary material for: GPR84 signaling promotes intestinal mucosal inflammation via enhancing NLRP3 inflammasome activation in macrophages
Source: Acta Pharmacol Sin. 2021 Dec 15;43(8):2042–54. doi: 10.1038/s41401-021-00825-y (PMC9343429; doi:10.1038/s41401-021-00825-y)

**Supplementary Information**

**GPR84 signaling promotes intestinal mucosal inflammation via enhancing NLRP3 inflammasome activation in macrophages**

Qing Zhang^1,2,#^, Linhai Chen^1,#^, Hui Yang^1,#^, Youchen Fang^1^, Siwei Wang^1,3^, Min Wang^1^, Qianting Yuan^1^, Wei Wu^4^, Yangming Zhang^5^, Zhanju Liu^4,*^, Fajun Nan^1,2,6,*^, Xin Xie^1,2,3,*^

^1^State Key Laboratory of Drug Research, the National Center for Drug Screening, Shanghai Institute of Materia Medica, Chinese Academy of Sciences, Shanghai, 201203, China

^2^School of Pharmaceutical Science and Technology, Hangzhou Institute for Advanced Study, University of Chinese Academy of Sciences, Hangzhou, 310024, China

^3^University of Chinese Academy of Sciences, No. 19A Yuquan Road, Beijing, 100049, China.

^4^Department of Gastroenterology, The Shanghai Tenth People’s Hospital, Tongji University, Shanghai, 200072, China

^5^Burgeon Therapeutics Co., Ltd., Shanghai, 201203, China.

^6^Yantai Key Laboratory of Nanomedicine & Advanced Preparations, Yantai Institute of Materia Medica, Shandong, 264000, China.

^#^These authors contributed equally to this work.

^*^Send correspondence to: Xin Xie, xxie@simm.ac.cn; Fajun Nan, fjnan@simm.ac.cn; or Zhanju Liu, liuzhanju88@126.com

**Supplementary Figure S1**

**
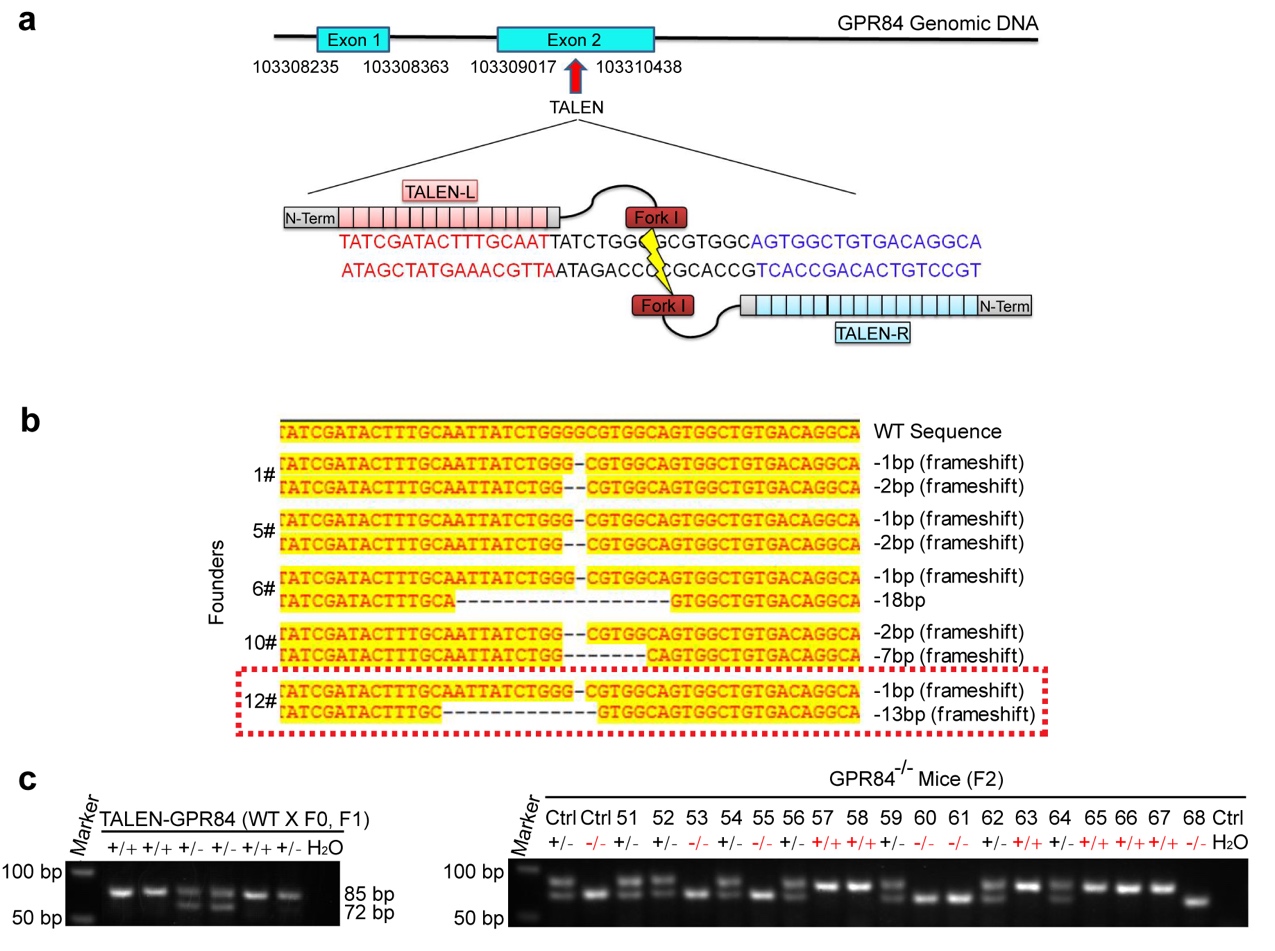
**

**Supplementary Figure S1.** TALEN-induced mutations in the GPR84 gene. (a) Schematic of GPR84 gene structure and TALEN binding sites. The left and right TALEN binding sites are shown in red and blue, respectively, and the Fork I site in the spacer is highlighted in yellow. (b) Representative sequencing results of different deletion mutations in F0 mice. Dashes indicate deleted bases and the numbers on the right side give the deletion sizes. (c, d) PCR analysis of genotype of F1 (c) and F2 (d) mice. All mice were confirmed by sequencing before further breeding.

**Supplementary Figure S2**

**
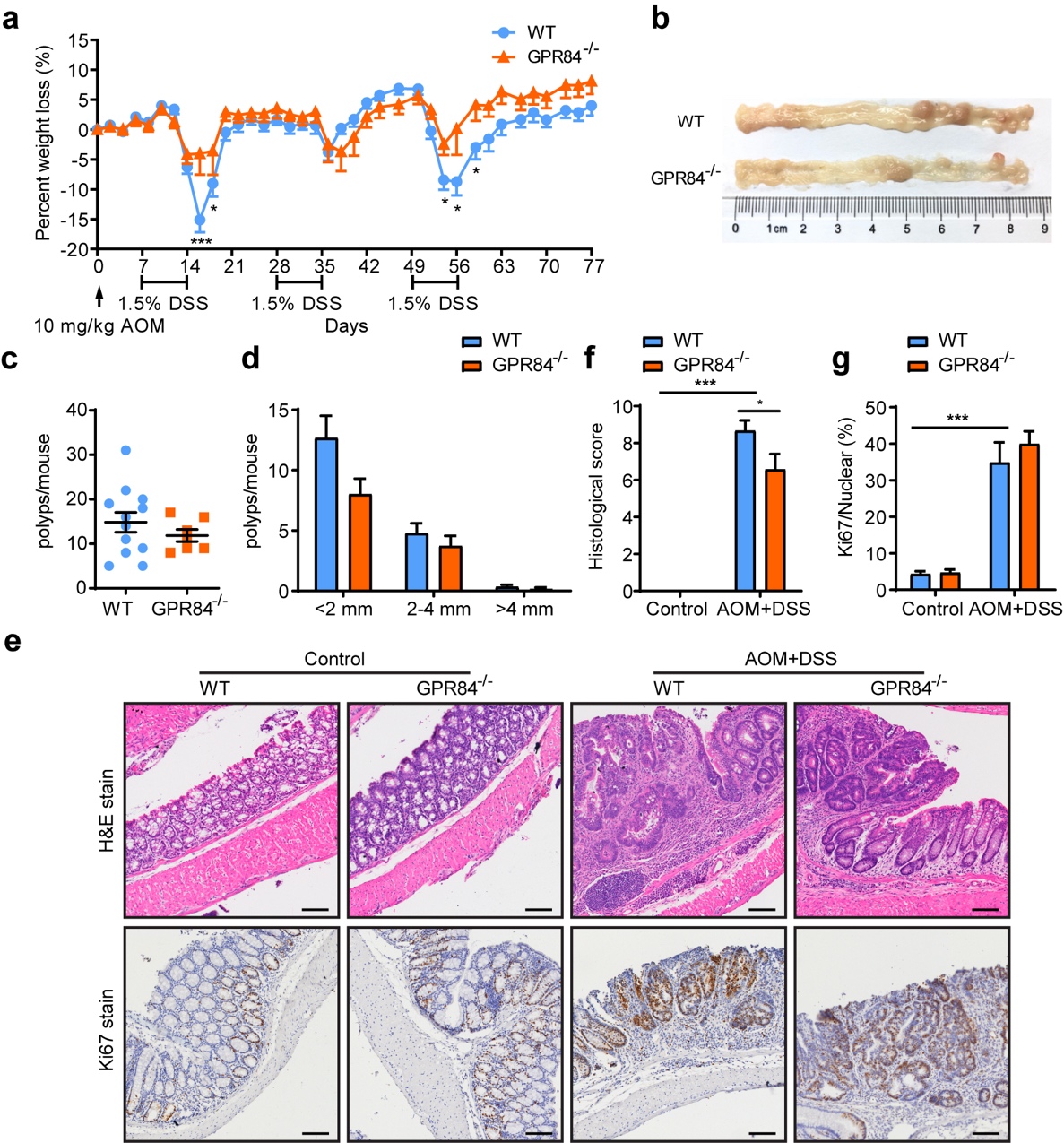
**

**Supplementary Figure S2.** Role of GPR84 in chronic colonic inflammation and inflammation-associated colon cancer. (a) Change in body weight of WT and GPR84^-/-^ mice subjected to AOM+DSS treatment (n>7). (b) Representative photograph of dissected colons from WT and GPR84^-/-^ mice on day 77 after AOM+DSS treatment. (c, d) The number (c) and size distribution (d) of colonic polyps induced by AOM+DSS treatment in WT and GPR84^-/-^ mice (n>7). (e, f, g) Representative images (e) of H&E and Ki-67 staining of colonic sections from control or AOM+DSS-treated (day 77) WT or GPR84^-/-^ mice (n>7, scale bar=100 μm), and statistical analysis of histological scores (f) and Ki-67^+^ cells per high-power field (g) (HPF, average of 3–6 different visual fields) in WT and GPR84^-/-^ mice (n > 7). Data are means ± SEM, *p < 0.05, ***p < 0.001 (unpaired student’s t test)

**Supplementary Figure S3**

**
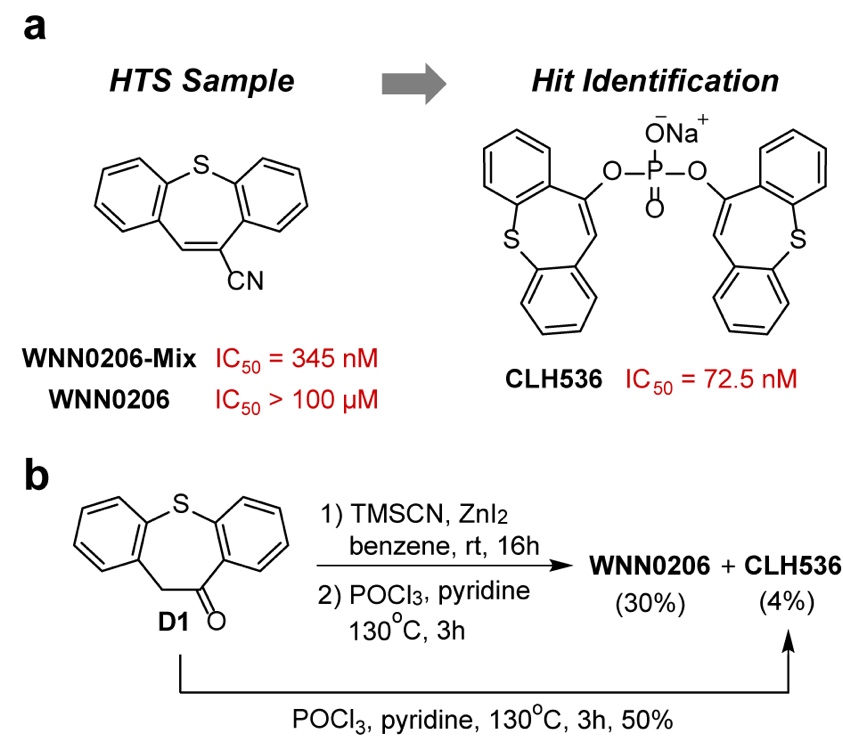
**

**Supplementary Figure S3.** (a) Chemical structures of CLH536 and WNN0206. (b) The synthesis of CLH536 and WNN0206.

**Supplementary Figure S4**

**
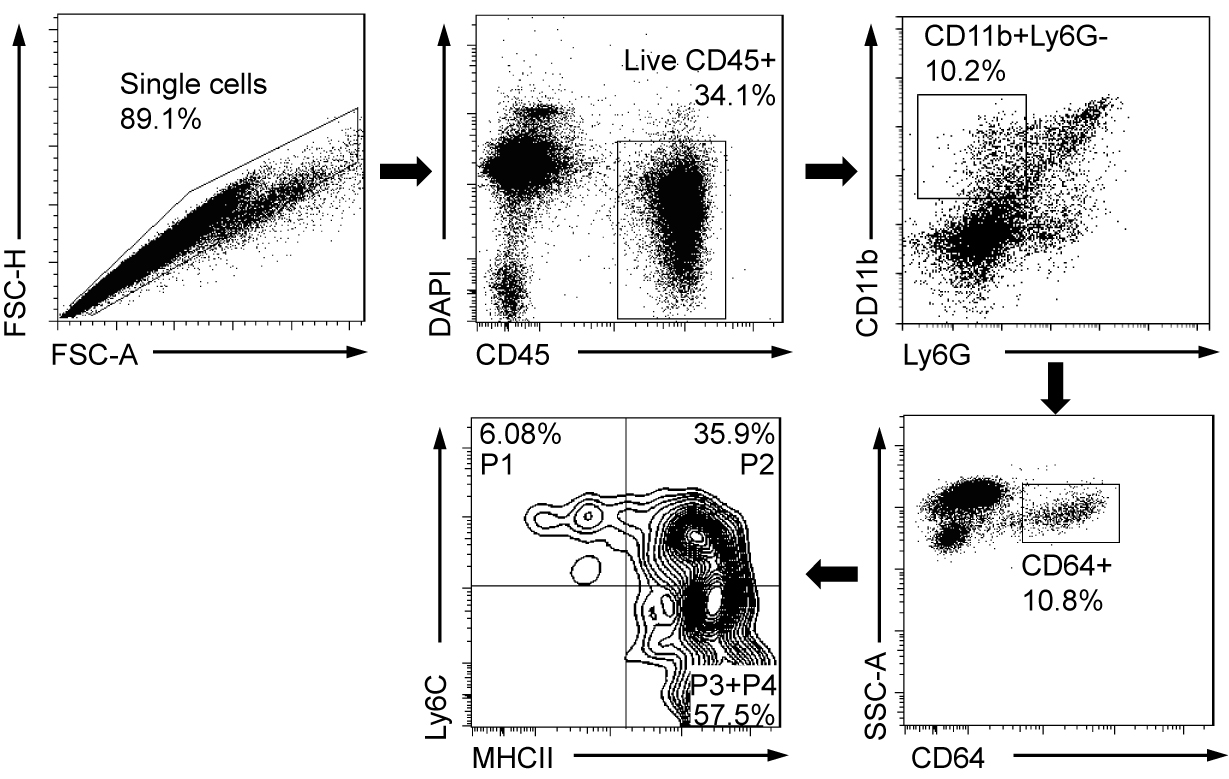
**

**Supplementary Figure S4.** Flow cytometry gating strategy for macrophage populations in colon from mice. Within the live CD45^+^ colonic lamina propria cells, a CD11b^+^Ly6G^-^ population was selected and from this gate the CD64^+^ population (macrophages) was further divided based on Ly6C and MHC class II markers. Three different macrophage populations can be discriminated (P1-4).

**Supplementary Figure S5**

**
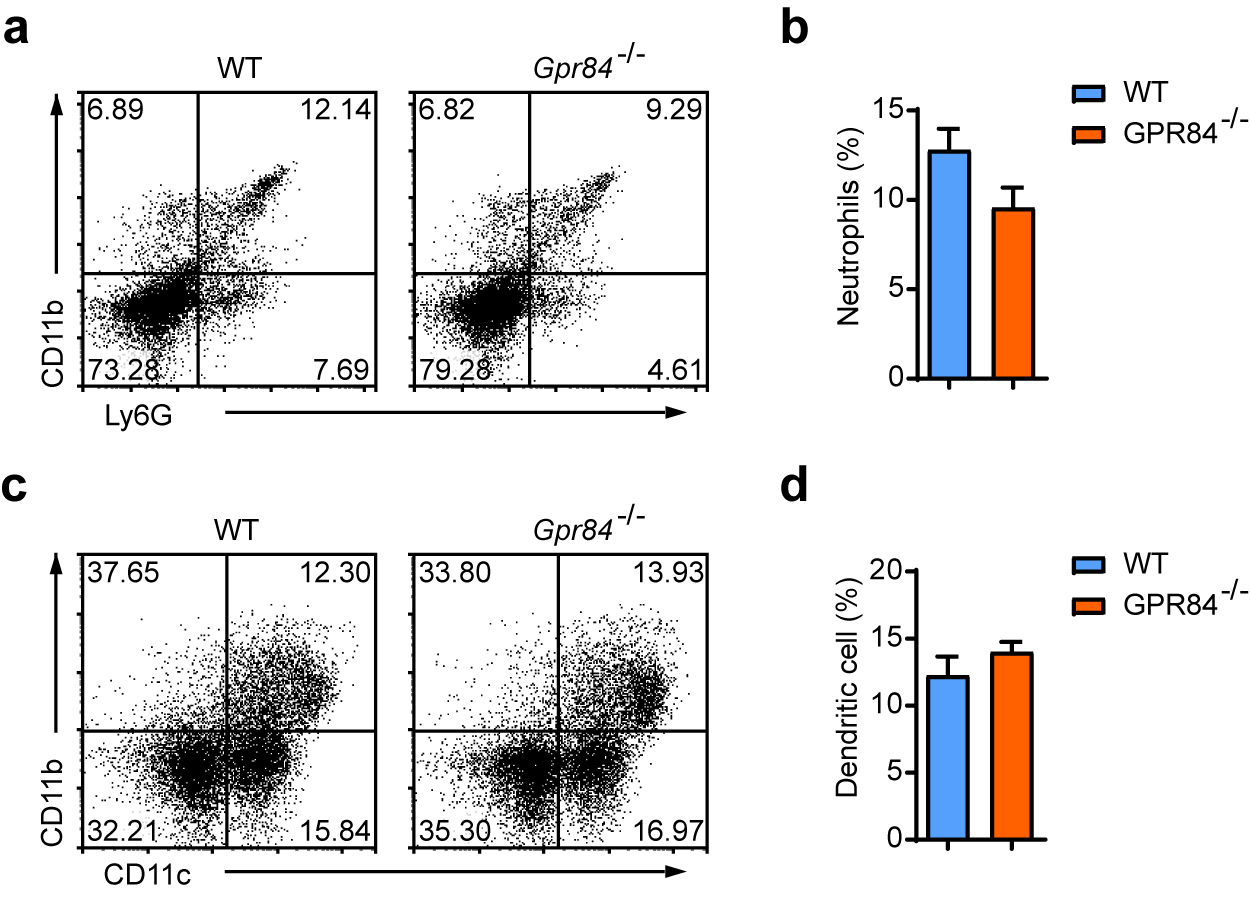
**

**Supplementary Figure S5.** Deletion of GPR84 shows limited effect on neutrophils and dendritic cells in the colonic lamina propria of DSS-induced mice. (a-d) Representative flow cytometry plots of CD11b^+^Ly6G^+^ neutrophil (a) and CD11b^+^CD11c^+^ DCs (c) in colonic lamina propria of DSS-induced WT and GPR84^-/-^ mice on day 7, followed by quantification of neutrophil (b) and DCs (d) populations (n=12-22 mice/group).

**Supplementary Figure S6**

**
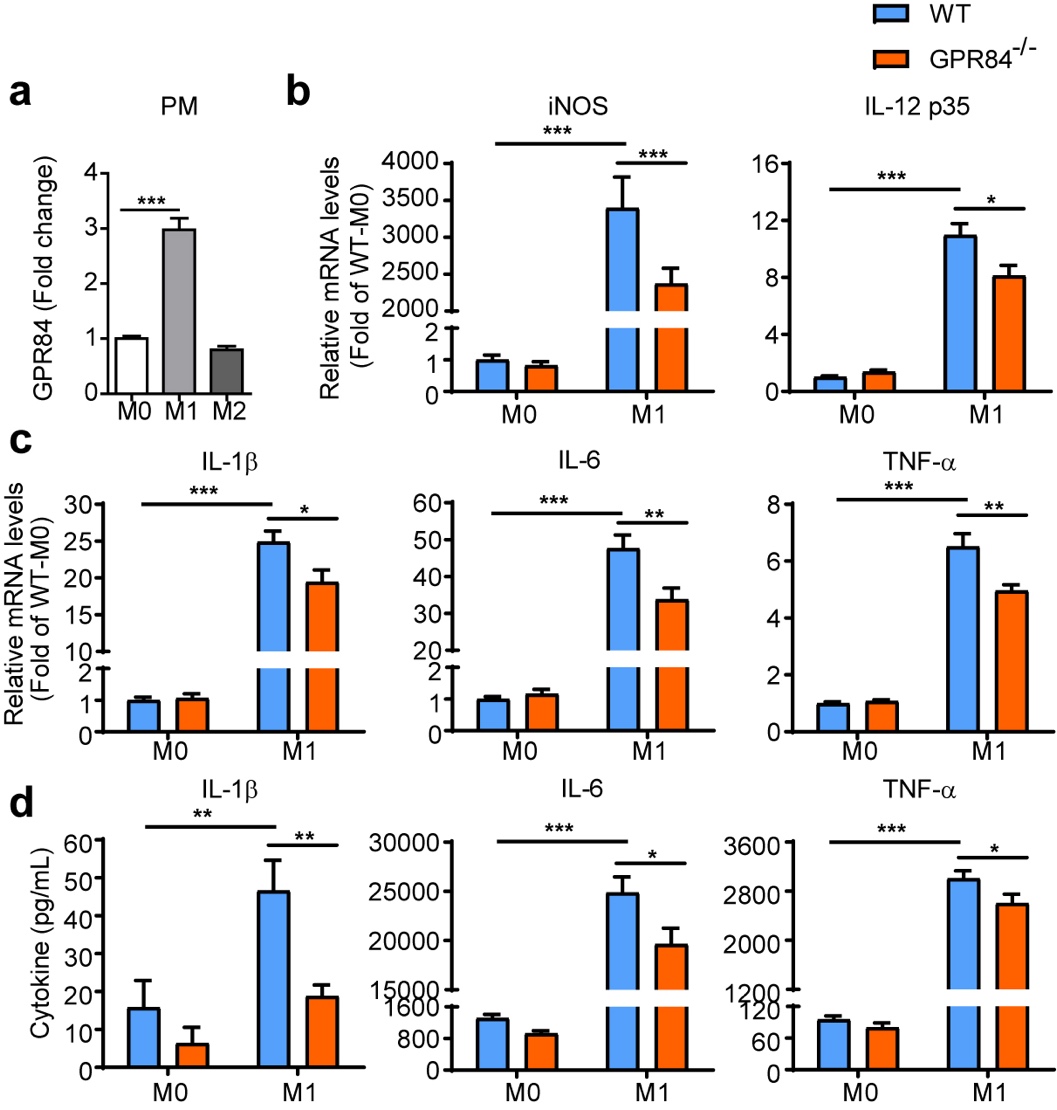
**

**Supplementary Figure S6.** GPR84 knockout reduces the proinflammatory M1 polarization of peritoneal macrophage (PM). (a) qRT-PCR analysis of GPR84 expression in unpolarized (M0), classically activated (M1), or alternatively activated (M2) PMs (n=4). (b) qRT-PCR analysis of iNOS and pro-inflammatory cytokine expression in PMs cultured in M0 or M1 conditions for 24 hr. Results were normalized to GAPDH expression in the same sample and then normalized to the control (WT M0 BMDM) (n=4). (c) ELISA analysis of cytokines in the supernatants of PMs cultured in M0 or M1 conditions for 24 hr (n=4). Data are expressed as means ± SEM. *p<0.05, **p<0.01, ***p<0.001 (unpaired student’s t test).

**Supplementary Figure S7**

**
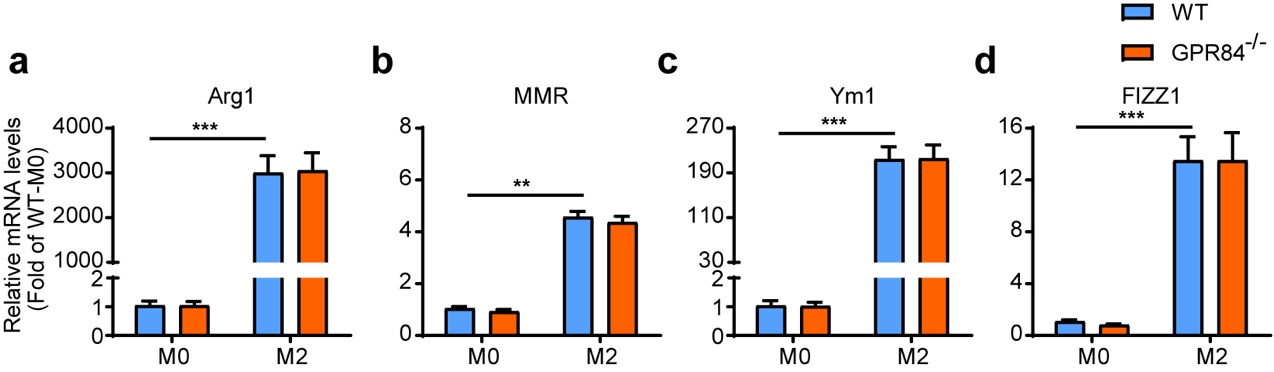
**

**Supplementary Figure S7.** GPR84 receptor deficiency does not affect anti- inflammatory M2 population in *in vitro* differentiation. (a-d) qRT-PCR analysis of anti-inflammatory cytokine expression in BMDMs cultured for 24 hr in M0 or M2 conditions. Results were normalized to GAPDH expression in the same sample and then normalized to the control (WT M0 BMDM) (n=4). Data are expressed as means ± SEM. **p<0.01, ***p<0.001 (unpaired student’s t test).

**Supplementary Table 1. Clinical Characteristics of IBD Patients**

|  | Con | CD (A/R) | UC (A/R) |
| --- | --- | --- | --- |
| **No. of patients** | 24 | 33 (20/13) | 32 (21/11) |
| Age (y) | 38.6 ± 13.4 | 32.0 ± 13.9 | 41.7 ± 15.1 |
| Gender |  |  |  |
| Male | 11 | 19 (12/6) | 17 (11/6) |
| Female | 13 | 14 (5/7) | 15 (10/5) |
| Disease duration (mo) |  | 38.3 ± 36.5 | 44.6 ± 38.6 |
| **Current therapy** |  |  |  |
| 5-aminosalicylates |  | 28 (15/13) | 29 (18/11) |
| Immunosuppressants |  | 0 | 0 |
| Biologics |  | 0 | 0 |
| Nutritional therapy |  | 5 (5/0) | 3 (3/0) |
| **Disease extent (UC)*** |  |  |  |
| E1 |  |  | 9 (7/2) |
| E2 |  |  | 11 (6/5) |
| E3 |  |  | 12 (8/4) |
| **Disease location (CD)*** |  |  |  |
| L1 |  | 7 (4/3) |  |
| L2 |  | 11 (6/5) |  |
| L3 |  | 15 (10/5) |  |
| L4 |  | 0 |  |

Abbreviations: A/R, active/remission; Con, healthy controls

CD, Crohn’s disease; UC, ulcerative colitis

*According to the Montreal classification system

**Supplementary Table 2. Oligonucleotides used for q-PCR analysis**

| **Gene** | **Forward primer (5'-3')** | **Reverse primer (5'-3')** |
| --- | --- | --- |
| **Mouse** | | |
| GPR84 | CTCCTGCTACCATGAGTCTGT | GTGCAGTAGAGTAGATCAGCCA |
| TNF-a | CTGAACTTCGGGGTGATCGG | GGCTTGTCACTCGAATTTTGAGA |
| IL-1b | GCAACTGTTCCTGAACTCAACT | ATCTTTTGGGGTCCGTCAACT |
| IL-6 | TAGTCCTTCCTACCCCAATTTCC | TTGGTCCTTAGCCACTCCTTC |
| iNOS | GTTCTCAGCCCAACAATACAAGA | GTGGACGGGTCGATGTCAC |
| IL-12-P35 | ACTCTGCGCCAGAAACCTC | CACCCTGTTGATGGTCACGAC |
| Arg1 | CTCCAAGCCAAAGTCCTTAGAG | AGGAGCTGTCATTAGGGACATC |
| Ym1 | GGGCATACCTTTATCCTGAG | CCACTGAAGTCATCCATGTC |
| MMR | CTCTGTTCAGCTATTGGACGC | CGGAATTTCTGGGATTCAGCTTC |
| FIZZ1 | GCCAGGTCCTGGAACCTTTC | GGAGCAGGGAGATGCAGATGAG |
| GAPDH | TCAACAGCAACTCCCACTCTT | ACCCTGTTGGTGTAGCCGTAT |
| **Human** | | |
| GPR84 | AGCTCCGTACCCGATTCAAC | ATGAGGAGGTAGCGTCCCAG |
| GAPDH | GGAGCGAGATCCCTCCAAAAT | GGCTGTTGTCATACTTCTCATGG |

**Supplementary Table 3. Specificity of CLH536 on GPCRs**

| Receptors | Calcium assay (nM) | | |
| --- | --- | --- | --- |
|  | Agonist (EC_50_) | Antagonist (IC_50_) |  |
| GPR84 | NR | 75.8±22.9 |  |
| GPR40 | NR | > 10 μM |  |
| GPR41 | > 100 μM | > 10 μM |  |
| GPR119 | NR | NR |  |
| GPR120 | NR | > 100 μM |  |
| α_2A_-AR | NR | NR |  |
| α_2B_-AR | NR | NR |  |
| α_2C_-AR | NR | NR |  |
| β_1_-AR | NR | NR |  |
| β_2_-AR | NR | NR |  |
| β_3_-AR | NR | NR |  |
| DRD1 | NR | NR |  |
| DRD2 | NR | NR |  |

NR, no response at concentrations up to 100 μM; Data are means ± SEM (n=3).

**Supplementary Information for Chemistry**

**General Procedure.** All the starting materials, reagents, and solvents were purchased from commercial suppliers and used without further purification. All non-aqueous reactions were run under an inert atmosphere (nitrogen or argon) with the rigid exclusion of moisture from reagents, and all reaction vessels were oven-dried. All the reactions were monitored by thin-layer chromatography (TLC), carried out on silica gel plates (HSGF 254, Yantai Jiangyou Chemical, Yantai, China). Spots were visualized under UV at 254 nm. Column chromatography (CC) was performed using silica gel (200–300 mesh, Qingdao Haiwan Specialty Chemicals Co. Ltd., Qingdao, China). Proton (^1^H) and carbon (^13^C) NMR spectra were recorded on a Bruker Avance III 500 spectrometer or a Varian Mercury VX300 Fourier transform spectrometer using deuterated DMSO (DMSO-*d*_6_) as the solvent. Chemical shifts were reported in δ (ppm) using the δ 2.50 signal of DMSO-*d*_6_ (^1^H NMR) and the δ 39.52 signal of DMSO-*d*_6_ (^13^C NMR) as internal standards. Abbreviations for signal coupling are as follows: s, singlet; d, doublet; t, triplet; td, triple doublet; dd, double doublet; m, multiplet. Coupling constants (*J*) are given in Hz. Low-resolution mass data were obtained on an Agilent 6110 Single Quadrupole LC/MS System. High-resolution mass data were obtained on a MICROMASS Q-Tof Ultima^TM^ spectrometer. The purity was determined by analytical HPLC chromatograms using Agilent 1200 series LC system equipped with a degasser, a quaternary pump, an autosampler, a column oven, and a diode array detector. Analytes were separated on a Zorbax SB C18 column (4.6 × 150 mm, 5 μm) and monitored at 260 nm at 25^o^C. Mobile phase: A = H_2_O (0.1% TFA), B = MeOH (0.1% TFA). Gradient 20% to 95% in 10 minutes with a flow rate of 1.0 mL/min. The following abbreviations for solvents and reagents are used: dimethylsulfoxide (DMSO), ethyl acetate (EtOAc), petroleum ether (PE).

Synthesis of compound **CLH536***^a^*

*^a^*Reagents and conditions: (a) KOH, Cu, H_2_O, reflux, 16h, 96%; (b) PPA, 100^o^C, 4h, 92%; (c) POCl_3_, pyridine, 100^o^C, 12h, 69%

**2-(2-(Phenylthio)phenyl)acetic acid (C1).** The title compound was prepared according to the reported procedure with some modifications (*J. Med. Chem.* **2010**, *53*, 7021–7034). To a solution of KOH (4.22 g, 75.3 mmol) in H_2_O (43 mL) was added benzenethiol (**B1**, 2.33 mL, 22.6 mmol), and the mixture was stirred at 50^o^C for 10 mins, and copper powder (0.41 g, 6.45 mmol) was added, followed by 2-iodo phenylacetic acid (5.00 g, 18.1 mmol). The reaction mixture was refluxed under a nitrogen atmosphere for 12 hrs. After cooling down to room temperature, the mixture was filtered through Celite, and the filtrate was washed with PE (3 × 20 mL) to remove the excess benzenethiol. Then the aqueous phase was acidified to pH = 2 with 6N HCl under vigorous stirring, and a white solid was precipitated. The solid was filtered, washed with cold water and dried under vacuum to give compound **C1** as a light yellow solid (4.50 g, yield 96%), which was used directly for the next step without further purification.

**Dibenzo[b,f]thiepin-10(11H)-one (D1).** Compound **C1** (4.50 g, 17.4 mmol) was mixed with polyphosphoric acid (PPA, 30 mL) and heated to 100^o^C under mechanical stirring. After 4 hrs, the mixture was poured into ice-water (200 mL) and stirred for 30 mins. Then the mixture was extracted with EtOAc (2 × 100 mL), and washed with H_2_O (30 mL), Sat Na_2_CO_3_ (30 mL) and brine (30 mL). After solvent removal in vacuo, the obtained residue was purified by silica gel column chromatography (PE/EtOAc = 50/1 ~ 25/1) to give product **D1** as a light yellow solid (3.62 g, yield 92%). ^1^H NMR (300 MHz, DMSO-*d*_6_): δ 8.06 (d, *J* = 8.0 Hz, 1H), 7.71 (d, *J* = 8.1 Hz, 2H), 7.64 – 7.51 (m, 2H), 7.51 – 7.35 (m, 2H), 7.30 (t, *J* = 7.7 Hz, 1H), 4.32 (s, 2H). MS (ESI positive): m/z [M+H]^+^ : 227.1.

**Sodium bis(dibenzo[b,f]thiepin-10-yl) phosphate (CLH536).** In a 10 mL round bottom flask, dibenzo[b,f]thiepin-10(11H)-one (**D1**, 1.00 g, 4.42 mmol) was dissolved in anhydrous pyridine (2.5 mL) at 60^o^C, followed by the addition of fresh distilled POCl_3_ (202 μL, 2.21 mmol). The reaction mixture was heated to 100^o^C and stirred overnight. After cooled to room temperature (r.t.), the reaction mixture was poured into cold 1N HCl/H_2_O (25 mL) and stirred for 5 mins. Then the mixture was extracted with EtOAc (3 × 10 mL), washed with saturated Na_2_CO_3_ (2 × 10 mL), dried over Na_2_SO_4_, filtered and concentrated. The residue was purified by flash chromatography over silica gel (DCM/MeOH = 15/1~ 10/1) to afford product **CLH536** as a white solid (0.823 g, yield 69%). ^1^H NMR (500 MHz, DMSO-*d*_6_): δ 7.74 (dd, *J* = 7.7, 1.3 Hz, 2H), 7.45 (t, *J* = 6.1 Hz, 4H), 7.38 (s, 2H), 7.35 (td, *J* = 7.5, 1.5 Hz, 2H), 7.32 – 7.27 (m, 4H), 7.25 (td, *J* = 7.4, 1.6 Hz, 2H), 7.19 (dd, *J* = 7.5, 1.0Hz, 2H). ^13^C NMR (126 MHz, DMSO-*d*_6_): δ 151.31 (d, *J* = 7.8 Hz), 139.13 (d, *J* = 6.5 Hz), 138.74, 134.07, 132.56, 131.97, 131.84, 130.08, 128.97, 128.32, 128.21, 128.08, 127.90, 117.09. HRMS (ESI): m/z calculated for C_28_H_18_NaO_4_PS_2_ – Na^+^ [M – Na^+^]: 513.0390, found 513.0405. Purity: 99.2% (HPLC).

**NMR Spectra**

Compound **D1** ^1^H NMR (300 MHz, DMSO-*d*_6_)


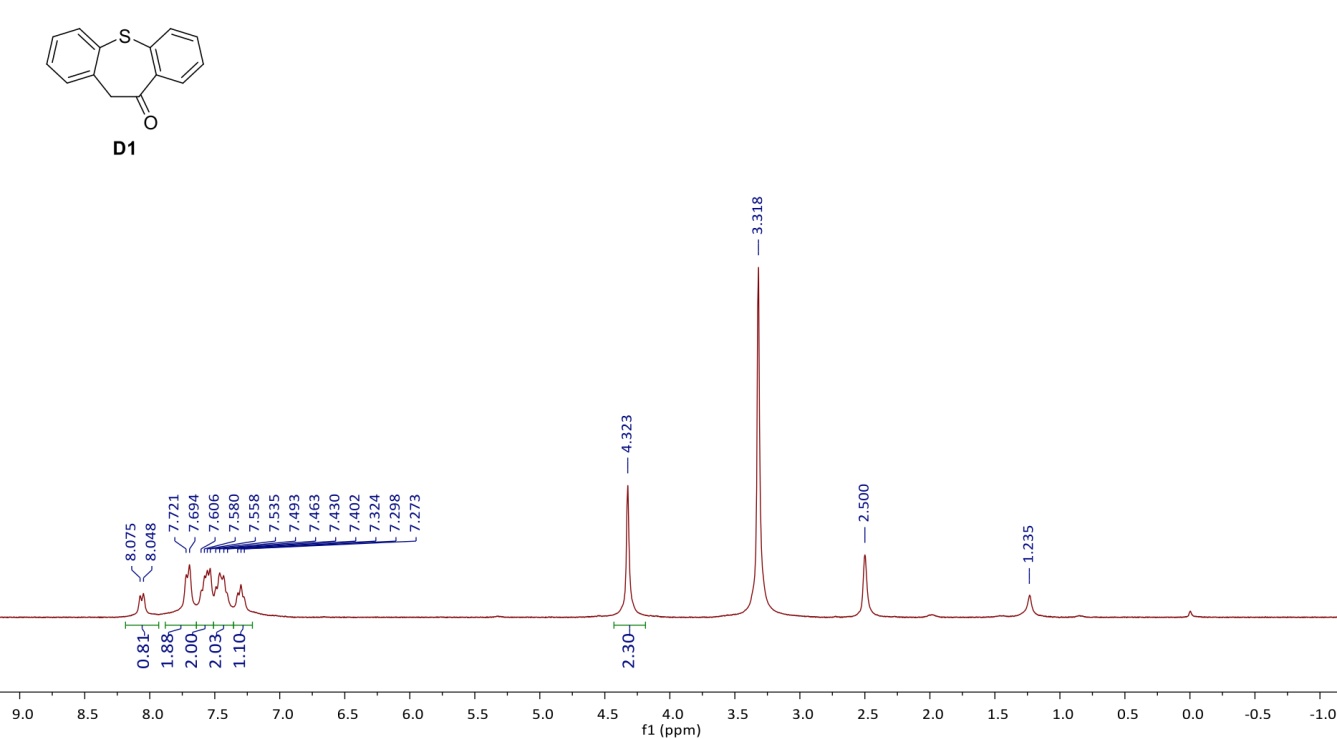


**CLH536** ^1^H NMR (500 MHz, DMSO-*d*_6_)

**
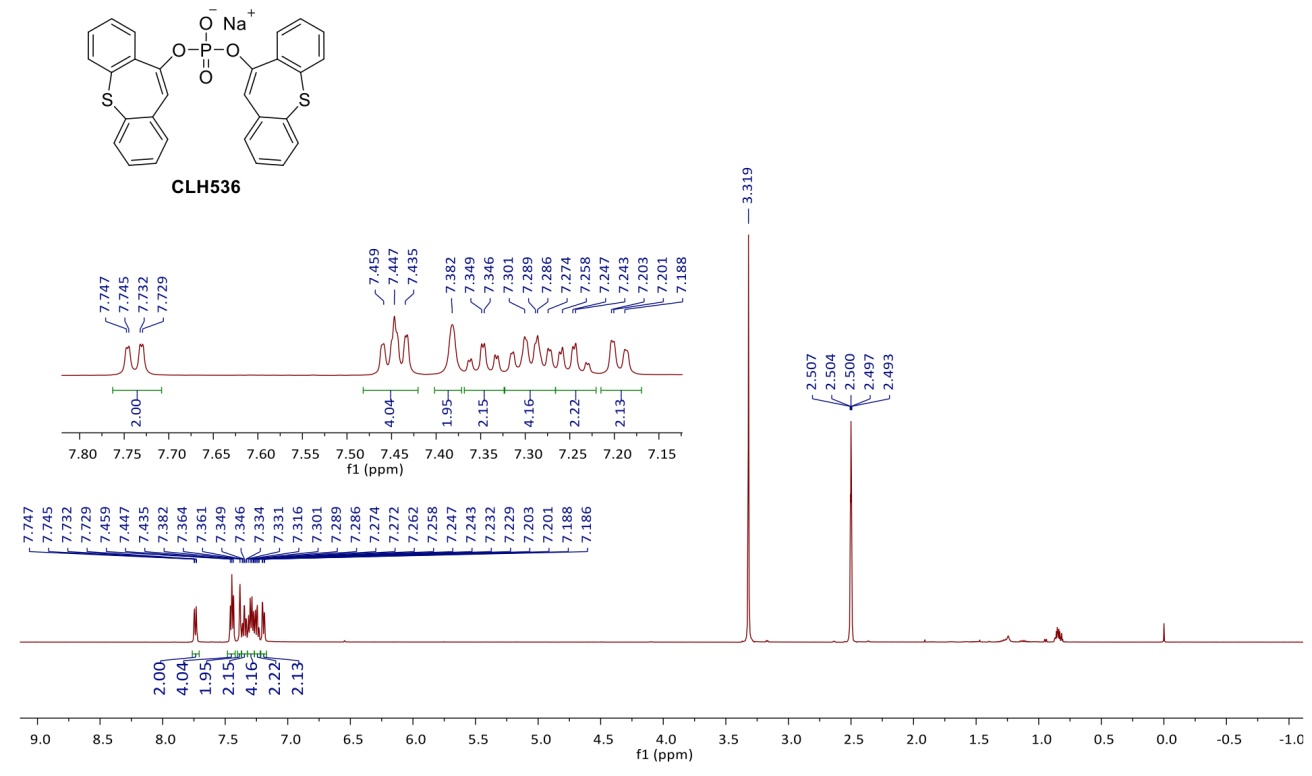
**

**CLH536** ^13^C NMR (126 MHz, DMSO-*d*_6_)


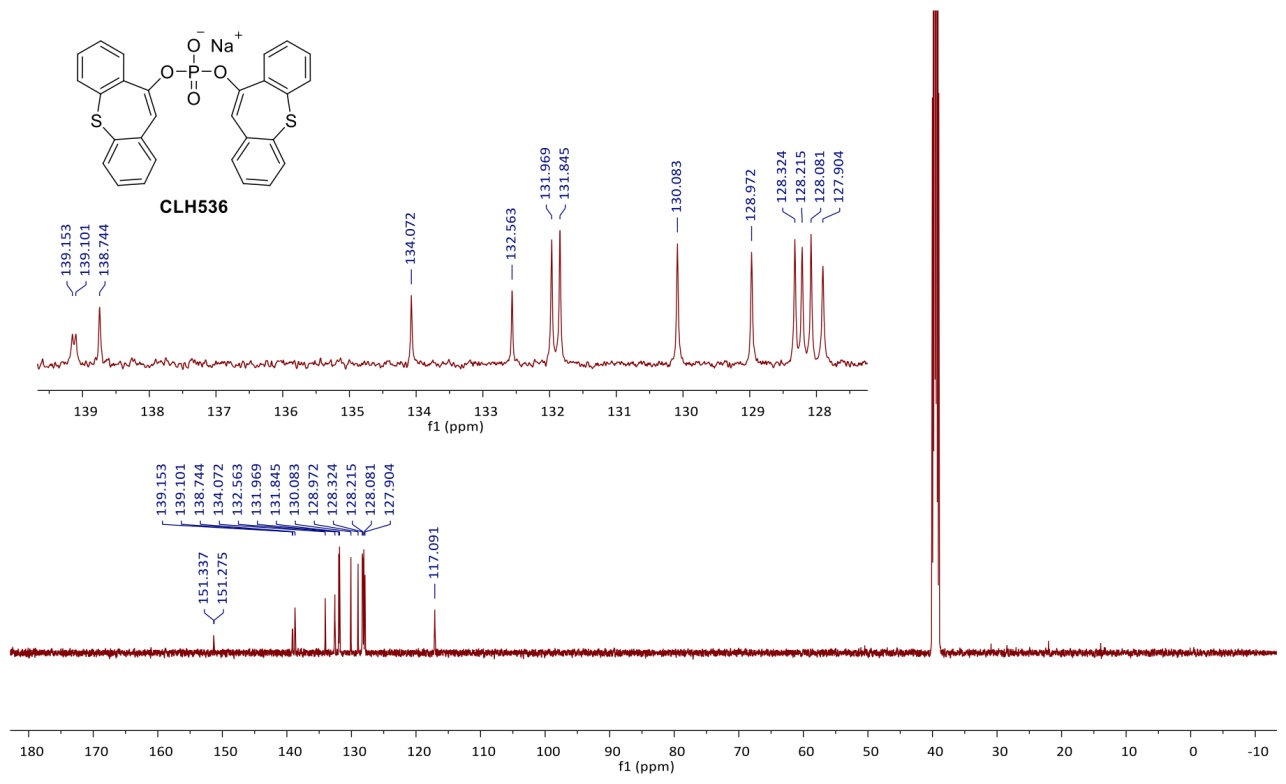

Supplement: Supplementary file 1 — Supplementary information [file 41401_2021_825_MOESM1_ESM.docx]
